# Dietary fibre blend improves gut health in patients with irritable bowel syndrome following a low FODMAP diet: a randomised, double-blind control trial

**DOI:** 10.1007/s00394-026-04113-5

**Published:** 2026-09-24

**Authors:** Ran Yan, Amanda Devine, Johnny Lo, Evania Marlow, Ian C. Dunican, Kanita Kunaratnam, Lesley Andrew, Claus T. Christophersen

**Affiliations:** 1 School of Medical and Health Sciences, Edith Cowan University, 270 Joondalup Drive, Joondalup, WA Australia; 2 Nutrition and Health Innovation Research Institute, Edith Cowan University, Joondalup, WA Australia; 3 School of Science, Edith Cowan University, Joondalup, WA Australia; 4 Joondalup Health Campus, Joondalup, WA Australia; 5 Centre for Sleep Science, School of Human Sciences, The University of Western Australia, Crawley, WA Australia; 6 School of Nursing and Midwifery, Edith Cowan University, Joondalup, WA Australia

**Keywords:** Irritable bowel syndrome, Dietary fibre, Gut microbiota, Short chain fatty acids, Quality of life

## Abstract

**Purpose:**

A diet low in FODMAP may negatively affect the gut microbiota of some Irritable Bowel Syndrome (IBS) patients, despite alleviating symptoms. This pilot study comprehensively explores the effects of a 3-week ur gut^®^ supplementation on gut microbiota, gut health, dietary intake, sleep, mental health and quality of life (QOL) in individuals with IBS following a low FODMAP diet.

**Methods:**

Adult participants (*n =* 26, 3 males) completed a randomised, double-blind controlled study. Participants were provided with either ur gut^®^ (*n =* 13) or placebo (*n =* 13) for 3 weeks after a 1-week baseline. At baseline and end of intervention, blood, 24-h faecal samples and 3-day weighed-food diaries were collected. Validated questionnaires were used to evaluate mental health, sleep, QOL and gut symptoms. Participants reported daily IBS symptoms and wore a wrist-based actigraphy to capture daily sleep.

**Results:**

During the intervention, both groups maintained baseline stable gut symptoms and sleep patterns. After the intervention, the ur gut^®^ group had lower energy (*P <* 0.05) and protein (*P <* 0.01) intake compared to the placebo group, and increased resistant starch intake (*P <* 0.001) compared to baseline. Changes in overall genus-level gut microbiota composition (*P <* 0.01), including *Ruminococcus E* (5.5-fold increase, *P <* 0.01), were observed. ur gut^®^ improved GI-specific anxiety (*P =* 0.01) and sexual subscale of QOL (*P <* 0.05).

**Conclusion:**

Compared with placebo, ur gut^®^ increased dietary fibre intake, improved gut microbiota and gastrointestinal-specific anxiety without negatively impacting sleep, despite no changes observed in faecal and blood biomarkers. This provides a dietary strategy to support gut health without compromising symptom control in IBS.

Australia and New Zealand Clinical Trial Registry http://www.anzctr.org.au/, ACTRN12620000032954.

**Graphical Abstract:**

**Figure:**
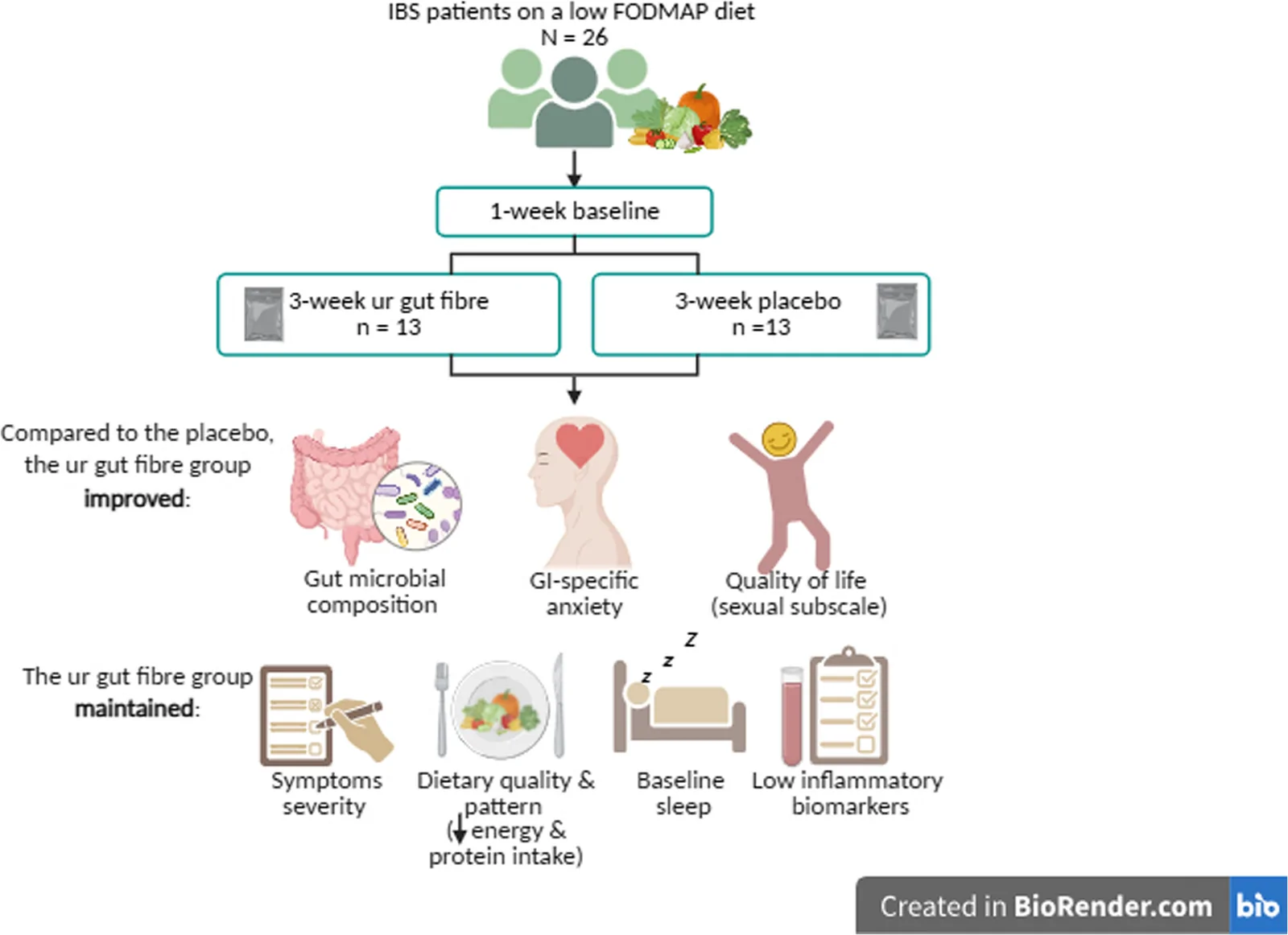

**Supplementary Information:**

The online version contains supplementary material available at https://doi.org/10.1007/s00394-026-04113-5.

## Introduction

Irritable Bowel Syndrome (IBS) affects around 5% of people globally [1], inflicting mental and life quality loss on individuals and imposing an economic burden on communities [2, 3]. A diet low in fermentable oligosaccharides, disaccharides, monosaccharides and polyols (FODMAP) has been demonstrated as efficient in IBS symptom management [4]. As a group of short-chain carbohydrates that occur naturally in varied foods, FODMAP is poorly absorbed in the small intestine and is rapidly fermented in the proximal colon, causing increases in water volume, osmotic activity, fermentation and gas production, which in turn leads to distention and symptom induction [5]. Following FODMAP consumption, similar physiological responses are involved in both IBS individuals and healthy people but due to visceral hypersensitivity, GI symptoms can be induced in IBS patients only [6, 7]. Moreover, IBS patients have been shown to have lower pain thresholds with higher perceptions of gut-related pain than the general population [8].

Gut health, encompasses, both subjective experience and objective aspects of gastrointestinal (GI) function, and the absence of symptoms does not necessarily indicate optimal gut health [9]. Despite symptomatic alleviation [10], a low FODMAP diet, however, has been suggested to have unfavourable impacts on gut microbiota, including: a reduction in total bacterial abundance [11]; prebiotic bacteria abundance, such as *Bifidobacteria* [12] ; butyrate-producing bacteria (*Faecalibacterium prausnitzii*); and gut health related bacteria (*Akkermansia muciniphila*) [13]. Moreover, despite symptom amelioration low FODMAP diets have not been shown to restore the dysbiotic gut microbiome of IBS, and can negatively impact the gut microbiota of healthy subjects [14, 15], while the clinical response can be dependent on the original microbiota signature in IBS adults patients [16, 17], and childhood IBS [18]. This was demonstrated in a longer-term study with 18 IBS patients who had completed a 3-phase FODMAP diet over a 12-month period showed that short chain fatty acid (SCFA) concentration was reduced compared to baseline, despite symptomatic improvement and unchanged *Bifidobacteria* abundance with a relative abundance at 1.2% − 1.3% [14]. However, a more recent meta-analysis found that the effects of a three- to four-week low FODMAP diet were largely limited to reductions in *Bifidobacteria* [12]. Nevertheless, there remains a need for research to explore strategies to maintain the symptom benefits achieved with a low FODMAP diet while simultaneously supporting or improving gut microbiota in this population.

Emerging research has shown the link between gut microbiota and IBS comorbidities, including problems in sleep [19] and mental health [20]. Moreover, gut microbiota has been indicated as a potential target for health-related improvement. Previous animal studies have found microbiota-derived butyrate to enhance sleep [21], where butyrate supplementation ameliorated damage in intestinal homeostasis induced by sleep deprivation [22]. SCFA are rapidly absorbed in the human colon, whereby SCFA levels decline along the large intestine, with approximately 5% of bacteria-derived SCFA being excreted in faeces [23], if excess amount is produced. Butyrate, one of the main SCFA, produced via fermentation of dietary fibre residue that enters the colon by local obligate anaerobic bacteria, is the preferred metabolic fuel for healthy colonocytes, providing approximately two-thirds of their energy (> 60%) [24]. Additionally, butyrate drives the energy metabolism of colonocytes towards mitochondrial beta-oxidation, which in turn consumes oxygen and maintains epithelial hypoxia [25, 26]. Butyrate can also modulate microbial tryptophan metabolites that appear to be dysfunctional in IBS patients, which are in turn linked to psychiatric comorbidities [27] and poorer sleep quality [28]. Therefore, existing evidence supports the pivotal role of microbiota-derived butyrate in functions such as sleep regulation and mental health outcomes [29].

Dietary fibre, as a key substrate for microbial fermentation and subsequent SCFA production, particularly butyrate [30], has been shown at lower levels in restrictive dietary patterns, such as a low FODMAP diet, at 10–17 g/d [14, 31]. This inadequate fibre intake has also been found in the IBS population [32], which is below Australian dietary recommendations [33]. In contrast, higher fibre intake has been positively associated with faecal SCFA. In addition, higher fibre intake also increases faecal output [34], and achieving a daily faecal output of approximately 150 g may reduce colon cancer risk through enhanced microbial fermentation and the production of beneficial metabolites, including butyrate [35, 36]. Given the heightened gastrointestinal sensitivity and altered visceral pain thresholds associated with IBS, identifying fermentable fibre sources that can be incorporated into the habitually low FODMAP diet while remaining well tolerated can be challenging. Resistant starch (RS) type 2 is not well tolerated in patients with IBS because of its high fermentable capacity and potential to exacerbate symptoms [37, 38]. Despite this limitation, RS has demonstrated many beneficial effects via reshaping the gut microbiome, including improvements in managing non-alcoholic fatty liver disease [39], facilitating weight loss [40], and reductions in inflammatory mediators [41]. Nevertheless, animal studies have shown that psyllium can shift the fermentation of high amylose resistant starch (HAMS, rich in RS2) further distally in the colon [42], suggesting that the mixture was likely to increase its tolerability. Therefore, this study proposed a novel intervention strategy involving a combination of RS2 and psyllium husk to support gut health in individuals with IBS who are following a low-FODMAP diet.

To summarize, a low FODMAP diet is an effective strategy for managing IBS symptoms; however, it may have unintended negative impacts on the gut microbiota. Therefore, this study investigated the feasibility and potential effects of a mixed-fibre intervention (ur gut^®^), consisting of RS2 and psyllium husk powder, administered to people with IBS following a personalised low-FODMAP diet. The primary aim was to determine whether this intervention is tolerable in people with IBS, supporting gut health without exacerbating gastrointestinal symptoms. In addition, little research has examined the interplay between gut microbiota, sleep, and mental health in the context of fibre-based dietary interventions for individuals with IBS following a low-FODMAP diet [43]. Therefore, the study also aimed to investigate the effects of ur gut® fibre on these outcomes and address this gap in the literature.

## Materials and methods

### Participants and sample size

Inclusion and exclusion criteria have been included in the protocol paper [44] and are detailed in the supplementary material. In summary, adult IBS patients (18–65 years old) were included if they self-reported stable symptoms a clinical diagnosis of IBS by a gastroenterologist or other medical professional, and had been on a low FODMAP diet for at least one month prior to the intervention. Participants were excluded if they were current smokers or had a known diagnosis of other gastrointestinal illness such as coeliac disease or inflammatory bowel disease.

The participant recruitment began in July 2020 in Perth, Western Australia. The initial sample size calculation proposed in the protocol indicated a total of 50 participants was required to detect a change in log SCFA concentration of 0.4, corresponding to a small-to-medium effect size of 0.2. However, the recruitment was significantly impacted by the COVID 19 pandemic, resulting in multiple lockdowns and reduced interest/engagement from the public. As a result, a revised sample size calculation was performed using G*Power version 3.1.9.2 [45]. The calculation was based on a within-between interaction design to minimally detect a medium effect size of 0.3 with 80% power and an α-error at 5%, whereby a minimum sample size of 24 was required. Accounting for a 15% attrition rate, the final minimum required sample size was 28 participants.

### Trial protocol

The study protocol has been published elsewhere [44], and methodological details are outlined in the supplementary methods. In summary, this study was designed as a randomised, double-blind, control trial, including a one-week baseline and a three-week intervention. At baseline (Timepoint 1, T1), participants were assessed for anthropometric measures, such as body weight (kg), waist and hip circumference (cm) and percentage of lean and fat mass (determined by BOD POD, an Air Displacement Plethysmograph). Fasting blood samples were collected to assess blood biomarkers of inflammation including Interleukin-6 (IL-6), IL-1β, Tumour Necrosis Factor alpha (TNF-α) and high-sensitivity C-reactive protein (hs-CRP). The baseline concentration and excretion amount of SCFA were examined from a 24-h faecal sample (all faecal output within the 24 h) collected on the last day of a 3-day weighed food diary. This was recorded by each participant to assess habitual dietary intake. The general diet quality was assessed using Aussie-Dietary Quality Index [46], detailed in the supplementary material. Varied validated questionnaires were used to assess IBS symptoms [47, 48], FODMAP intake [49], sleep [50–54], mental health [55, 56], QOL [57, 58] and physical activity [59], at T1 and at Timepoint 2 (T2) - the end of the interventional period, as detailed in the study protocol [44].

After the baseline week, participants were randomly assigned to one of two groups, either the ur gut^®^ fibre powder or placebo (maize flour without resistant starch with psyllium husk), for a period of three weeks. The intervention of ur gut^®^ fibre consists of HAMS and psyllium husk (International Patent Application (WO/2025/007187)). To ensure that any observed intervention effects were not due to psyllium husk, the placebo incorporated an identical psyllium husk component in the same amount. Both groups increased their dose from 5 g to 40 g/d over 11 days until the full dose was consumed for a further 10 days (40 g /d), as described in the study protocol [44]. A longer adaption period provided participants with additional time to adjust to the increased fibre intake relative to their habitual diet, which helped minimize any GI discomfort and risk of triggering IBS symptoms, thereby enhancing adherence to the intervention. For every 100 g of ur gut^®^, it provides 67 g carbohydrate consists of 56 g dietary fibre (36 g resistant starch) and 11 g other carbohydrate, as well as 1 g protein and less than 1 g of fat. Education about consumption, calculation of compliance and specimen collection and analysis are detailed in the supplementary material. A wrist-actigraphy monitor, a Readiband™ v5 (Readiband, Fatigue Science Inc., Canada) (validated [60]), was provided to each participant to capture daily sleep data. Throughout the four weeks, participants recorded their daily symptoms and notes via an online checklist. In addition to online daily intake records, to monitor compliance, the research team maintained regular follow-up contact and required participants to return unused sachets at the final visit (T2), where the assessments conducted at T1 were repeated. The primary outcome was severity of IBS symptoms, with other analyses listed as secondary outcomes.

The study was approved by the Human Research Ethics Committee of Edith Cowan University (2019-00619-YAN). The study was registered with the Australia and New Zealand Clinical Trial Registry (ACTRN12620000032954). All participants consented before the commencement of the study. All authors had access to the study data and reviewed and approved the final manuscript.

### Statistical methods

The per-protocol analysis was undertaken using data from participants who completed the study, whereby non-compliant participants were excluded. Linear Mixed Modelling was used to determine both within- and between-group differences in health outcomes via the group x time interaction effect and the main effects (if the interaction is not significant), with BMI and age included as covariates. Gender was not included due to the small number of male participants (*n =* 3). Post-hoc tests were conducted if a significant effect was detected. The significance level was set at *P ≤* 0.05. Furthermore, false discovery rate (FDR) correction was performed on all raw *P*-values to minimise false positive results, and an effect was noted as marginal when the raw *P ≤* 0.05, but the FDR *P >* 0.05. For gut microbiota, a combination of R, Primer 7 and Permutation Multivariate Analysis of Variance (PERMANOVA) + (PRIMER-E, Plymouth), was used to analyse bacterial community structure at three taxonomic levels; phylum, genus and Amplicon Sequencing Variant (ASV). The relative abundance data were square root transformed and a Bray-Curtis dissimilarity matrix was calculated, followed by the deployment of Principal Coordinates Analysis (PCoA) to visualise findings of the three taxonomic levels at T1 and T2. For determination of significant associations at baseline and longitudinal dissimilarities between the two groups, PERMANOVA and dissimilarity percentage (SIMPER) were conducted. Distance-based linear models (DistLM) and distance-based redundancy analysis (dbRDA) were used to integrate gut microbial findings with all other relevant aspects. Further details of the statistical analyses are provided in the supplementary methods.

## Results

### Participants

Overall, 30 participants consented to the study. In the placebo group, one female withdrew before being provided with the placebo. Another female withdrew on day 8 of placebo administration due to self-reported feelings of unwell, and one was lost to follow-up after administration. In the ur gut^®^ group, one female participant stopped consuming the supplement on day 8 due to self-reported nausea. These data were not included in any of the analyses. Therefore, a total of 26 participants (87%) completed the study and were included in the analysis (Supplementary results, Supplementary Fig. 1), which fulfilled the minimum required sample size of 24 participants. The mean age of participants was 37 ± 13 years, whereby 88% (*n =* 23) were female, with no between-group difference in demographic characteristics and IBS subtypes (Supplementary Table 1). No change was noted in anthropometrics and physical activity between T1 and T2 (Table 1). Compliance for the placebo group (94.8 ± 5.5%) was higher than the ur gut^®^ group (89.0 ± 6.5%) (*P <* 0.05), and both groups met the a priori compliance criteria (> 80%).

**Table 1:** Results of anthropometry and physical activity by group and timepoint

Anthropometry and physical activity	Group × Time Interaction *P*	ur gut^®^ Group, *n =* 13 Mean (SE)			Placebo Group, *n =* 13 Mean (SE)			Between-group *P*
		T1	T2	T2-T1	T1	T2	T2-T1	
Bodyweight, kg	0.057	65.0 (3.5)	64.9 (3.5)	-0.1 (0.3)	62.9 (3.5)	63.6 (3.5)	0.7 (0.3)¹	0.735
BMI, kg/m²	0.058	23.0 (0.9)	22.9 (0.9)	0.0 (0.1)	22.8 (0.9)	23.1 (0.9)	0.2 (0.1)²	0.995
Waist circumference, cm	0.629	74.3 (2.8)	74.6 (2.8)	0.3 (0.5)	76.2 (2.8)	76.9 (2.8)	0.7 (0.5)	0.600
Hip circumference, cm	0.203	99.2 (2.2)	99.0 (2.2)	-0.2 (0.5)	98.7 (2.2)	99.4 (2.2)	0.8 (0.5)	0.974
Waist/Hip ratio	0.359	0.7 (0.0)	0.8 (0.0)	0.0 (0)	0.8 (0.0)	0.8 (0.0)	0.0 (0.0)	0.496
Fat percent, %	0.610	24.9 (2.4)	24.4 (2.4)	-0.5 (0.5)	29.3 (2.4)	29.2 (2.4)	-0.1 (0.5)	0.180
Fat-free mass percent, %	0.610	75.1 (2.4)	75.6 (2.4)	0.5 (0.5)	70.7 (2.4)	70.8 (2.4)	0.1 (0.5)	0.180
Fat mass, kg	0.333	16.3 (2.1)	16.0 (2.1)	-0.3 (0.4)	18.7 (2.1)	18.9 (2.1)	0.2 (0.4)	0.371
Fat-free mass, kg	0.421	48.6 (2.8)	48.9 (2.8)	0.3 (0.4)	44.1 (2.8)	44.9 (2.8)	0.8 (0.4)	0.300
Body volume, L	0.236	62.3 (3.5)	62.5 (3.5)	0.1 (0.4)	60.9 (3.5)	61.6 (3.5)	0.7 (0.4)	0.817
Body density, kg/L	0.751	1.0 (0)	1.0 (0)	0.0 (0)	1.0 (0)	1.0 (0)	0.0 (0)	0.189
Systolic blood pressure, mm Hg	0.897	105 (4)	102 (4)	-3 (2)	109 (4)	107 (4)	-2 (2)	0.357
Diastolic blood pressure, mm Hg	0.416	70 (3)	69 (3)	0 (2)	74 (3)	71 (3)	-3 (2)	0.314
Heart rate, beats per minute	0.326	61 (3)	64 (3)	3 (2)	67 (3)	67 (3)	1 (2)	0.164
International Physical Activity Questionnaire								
Total MET ³, min/w	0.887	3477 (936)	3772 (954)	295 (-708)	2103 (936)	2257 (936)	153 (-684)	0.255
Vigorous intensity MET, min/w	0.069	1359 (406)	875 (417)	-484 (-398)	487 (406)	1059 (406)	572 (-385)	0.505
Moderate intensity MET, min/w	0.442	959 (371)	1613 (385)	654 (-427)	436 (371)	624 (371)	188 (-415)	0.099
Walking, min/w	0.242	1159 (444)	1362 (459)	203 (-484)	1180 (444)	574 (444)	-607 (470)	0.482

*T* Timepoint, *SE* Standard Error, *BMI* Body mass index, *Fat-Free Mass* Fat-free mass is everything except fat, including muscle, water, bone, and internal organs. For the anthropometric data, Linear Mixed Modelling (LMM) was used to assess differences between- and within-groups, without any adjustment. For physical activity data, LMM was used to assess differences between- and within-groups with BMI = 22.9049, and age = 37.27 included as covariates in the model

^1^*P =* 0.017

^2^*P =* 0.019, Both > 0.05 after False discovery rate adjustment

^3^Results reported as MET, unit was minutes per week, exercise equivalent minutes (MET minutes) weekly

### IBS symptoms and bowel habits

The severity of symptoms was assessed by two questionnaires: the Gastrointestinal Symptom Rating Scale for IBS [47] and the Irritable Bowel Syndrome-Symptom Severity Scale (IBS-SSS) [48]. Both groups had a moderate level of symptoms and did not change over the intervention (Table 2). Likewise, no interaction effect was found in daily GI symptom scores throughout the 4-week study (Supplementary Table 2).

Whilst the effect of intervention/placebo on bowel habits were examined longitudinally, no interaction effects were observed for stool weight and type (self-rated using Bristol Stool Chart [61]) or the number of bowel movements (an average of the last three days of each timepoint, same period as the 3-day food diary) (Supplementary Table 3).

**Table 2:** Results of IBS symptom questionnaires by group and timepoint

Questionnaire	Group × Time Interaction *P*	ur gut^®^ Group, *n =* 13 Mean (SE)			Placebo Group, *n =* 13 Mean (SE)			Between-group *P*
		T1	T2	T2-T1	T1	T2	T2-T1	
IBS-SSS	0.941	238.5 (22.7)	232.1 (22.7)	-6.4 (27.8)	272.9 (20.9)	265.0 (20.9)	-7.9 (25.6)	0.321
GSRS-IBS	0.599	49.2 (3.4)	45.0 (3.5)	-4.2 (3.9)	51.2 (3.4)	44.1 (3.4)	-7.1 (3.8)	0.891
Pain	0.892	7.5 (0.7)	6.7 (0.7)	-0.8 (0.8)	8.3 (0.7)	7.7 (0.7)	-0.6 (0.7)	0.296
Bloating	0.455	13.8 (1.1)	12.7 (1.2)	-1.1 (1.2)	14.3 (1.1)	12.0 (1.1)	-2.3 (1.1)	0.946
Constipation	0.876	6.5 (0.9)	5.9 (0.9)	-0.6 (0.8)	6.7 (0.9)	6.0 (0.9)	-0.8 (0.8)	0.878
Diarrhoea	0.327	14.0 (1.4)	12.8 (1.5)	-1.1 (1.8)	16.4 (1.4)	12.7 (1.4)	-3.7 (1.8)¹	0.458
Early satiety*	0.536	7.5 (0.9)	7.0 (0.9)	-0.5 (0.9)	5.4 (0.9)	5.7 (0.9)	0.3 (0.9)	0.130

*T* Timepoint, *SE* Standard error, *IBS-SSS* Irritable Bowel Syndrome-Symptom Severity Scale, *GSRS-IBS* Gastrointestinal Symptom Rating Scale for IBS; Linear Mixed Modelling was used to assess differences between- and within-groups with BMI = 22.9049,.and age = 37.27 included as covariates in the model. *P*-values reported are unadjusted

*Early satiety refers to if the fullness feeling appears shortly after meal and long after eating

^1^*P =* 0.05, False Discovery Rate *P >* 0.05

### Dietary outcomes (FODMAP, Macronutrients)

At T1, no between-group difference was noted for all dietary outcomes, except for energy from carbohydrate (in percentage), which was higher in the placebo group than the ur gut^®^ group (40.6 ± 8.9% vs. 31.5 ± 7.2%, *P <* 0.01, FDR-*P <* 0.05).

**Fig. 1:**
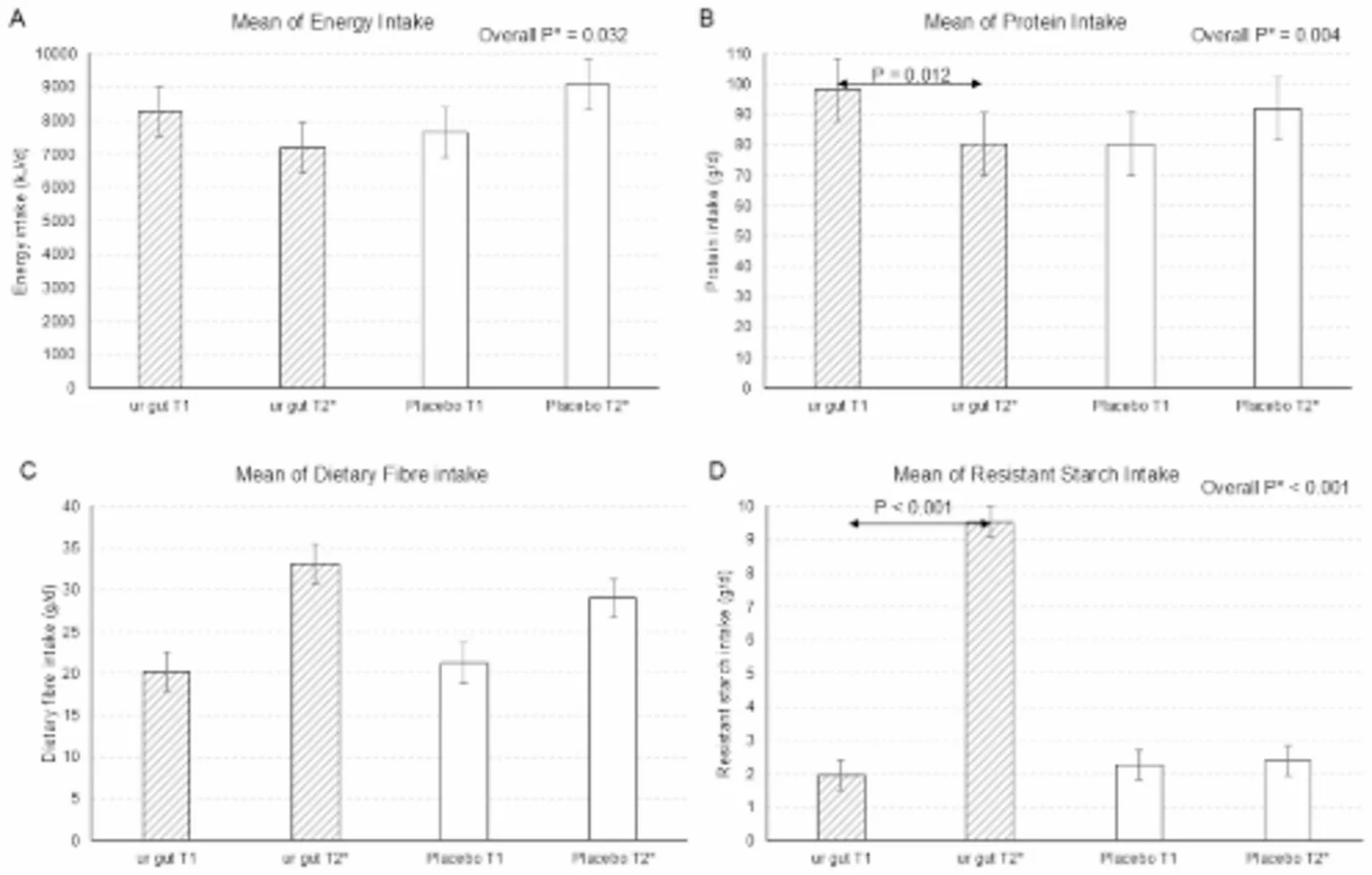
Comparison of intake of nutrients (energy, protein, fibre and resistant starch) by group and timepoint. **A** Overall energy intake; **B** Protein intake; **C** Dietary fibre intake (Dietary fibre intake exclusive of Resistant Starch intake); **D** Resistant starch intake. T, Timepoint. * indicates nutrients from both diet and supplement

Across the whole study period, there was no Group x Time interaction effect for any dietary outcomes, including Dietary Quality Index (DQI) and FODMAP intake, where these dietary markers remained stable in the respective groups (Supplementary Table 4). However, an interaction effect was observed for intakes of energy and protein (*P =* 0.032 and *P =* 0.004 respectively; both FDR *P <* 0.05). Both showed lower intakes in the ur gut^®^ group at T2* (T2* indicates values including both diet and supplement) in contrast to increases in the placebo group (Fig. 1A and B, Supplementary Table 4).

A marginal interaction effect (*P <* 0.05, FDR *P >* 0.05) was observed in habitual dietary fibre derived from diet only, with a decrease in the ur gut^®^ group at T2 compared to the habitual intake at T1; however, this reduction was offset by the ur gut^®^ supplement, leading to an overall increase in fibre intake at a total of 33.1 ± 2.4 g/d at T2 (Fig. 1C, Supplementary Table 4), which met daily Australian recommended levels for males (30 g/d) and females (25 g/d). A Group x Time interaction effect for RS was noted (*P <* 0.001, FDR *P <* 0.05), with the ur gut^®^ group increasing their intake of resistant starch to 9.5 ± 0.3 g/d at T2* from their T1 level at 2.0 ± 0.3 g/d (*P <* 0.001) (Fig. 1D, Supplementary Table 4). This reached the average intake level of Australian adults at 9.5 g/d [62], although only half of the suggested amount of 20 g/d [63]. Supplementary Fig. 2A–D shows individual participant changes in intake between T1 and T2 across each group, corresponding to Fig. 1A–D.

### Sleep

Participants exhibited normal sleep duration, sleep latency and sleep efficiency measured daily across the baseline week, according to the National Sleep Foundation’s recommendations [64], whereby no change was observed during the intervention in either group in relation to objective sleep measures (Supplementary Fig. 3). A marginal interaction effect was observed for Time at Sleep Onset (*P <* 0.05, FDR *P >* 0.05), where the placebo group delayed their sleep onset time, whilst the ur gut^®^ group maintained their sleep onset time throughout the study (Supplementary Fig. 3B). Similarly, no effects were observed in sleep questionnaires (Supplementary Table 5). These sleep outcomes indicated that sleep patterns and behaviours were stable in the ur gut^®^ group over the 4-week study.

### Mental health and quality of life

Suggestive evidence of an interaction effect was observed for the Visceral Sensitivity Index (VSI) [56] (*P <* 0.05, FDR-adjusted *P >* 0.05), where the ur gut^®^ group had a reduction of 7.4 ± 2.6 units at T2, whilst VSI remained steady for the placebo group (Supplementary Table 6). No interaction effects occurred on measures of the Depression Anxiety Stress Scales (DASS21) [55] (Supplementary Table 6). Two genera, *Alistipes* and *Coprococcus*, showed a positive correlation with VSI score (r_p_= 0.750 and 0.743, *P <* 0.001, respectively).

A marginal interaction effect was found on the sexual subscale of the IBS- QOL [57] (*P <* 0.05, FDR-adjusted *P >* 0.05), where the ur gut^®^ group had a within-group increase of 12.5 ± 4.9 (*P <* 0.05), whilst there was no notable change for the placebo group (Supplementary Table 7). No other changes were noted for all remaining seven subscales of IBS-QOL (Supplementary Table 7).

### Faecal SCFA and blood biomarkers

No interaction effects were shown on the concentration and excretion amount of SCFA in 24-hour stool samples (Supplementary Table 8). However, changes in faecal output and SCFA were found, when dividing both groups according to their T1 faecal output lower or greater than 150 g/d, a faecal index associated with improving fermentation products in relation to colon cancer risk [35, 36]. At T2, the ur gut^®^_LOW_ subgroup (*n =* 5, T1 faecal output < 150 g/d) increased the faecal butyrate concentrations and excretion by 51% and 289% of their T1 level (both *P <* 0.05, FDR *P >* 0.05), where their mean faecal output also increased from 78 to 162 g/d. The placebo _LOW_ subgroup (*n =* 6) also observed a 41% increase in stool weight (*P <* 0.05) without change in faecal butyrate (detailed in the Supplementary Results and Supplementary Tables 9 and 10).

Blood biomarkers of inflammation status were collected, but most of the results for IL-1β and TNF-α were below detection limits, leading to insufficient sample sizes for further analysis. For IL-6 and hs-CRP, no changes were observed (Supplementary Table 11).

### Gut microbiota

**Fig. 2:**
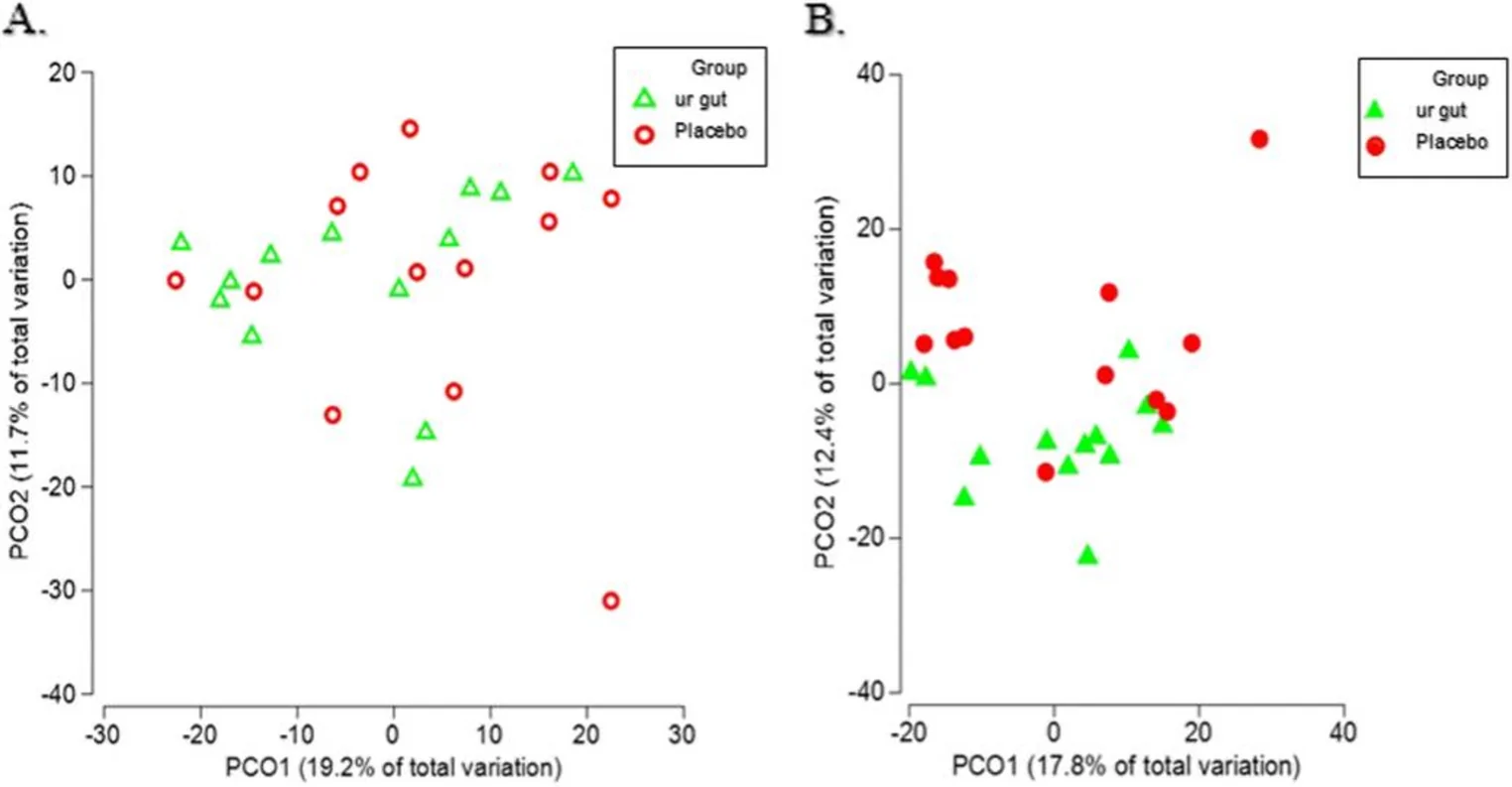
Principal coordinate analysis (PCoA) at the genus level of gut microbiota by group and timepoint. **A** PCoA of gut microbiota at the genus-level at T1. The variation in the genus community is best captured by the first two dimensions, PCO axes 1 & 2, which explain 30.9% of the variability between groups. **B** Genus-level gut microbiota of the two groups was significantly different at T2 (*P =* 0.0082). PCO plot suggests the variation in the genus community structure is best captured by the two dimensions, PCO axe 1 & 2 which explains 30.2% of the variability between groups at T2

PERMANOVA analysis did not show microbial composition changes at the phylum level (*P >* 0.05). However, overall microbial composition was altered at the genus level (*P =* 0.003), where between-group differences were observed at T2 (*P <* 0.01, Fig. 2B), despite no Group × Time interaction effect. Similarly, at ASV level, no between-group difference at T1 (*P >* 0.05) was observed; however, after the intervention, the microbial composition of the two groups differed (*P <* 0.01) (Supplementary Fig. 4A and B).

**Fig. 3:**
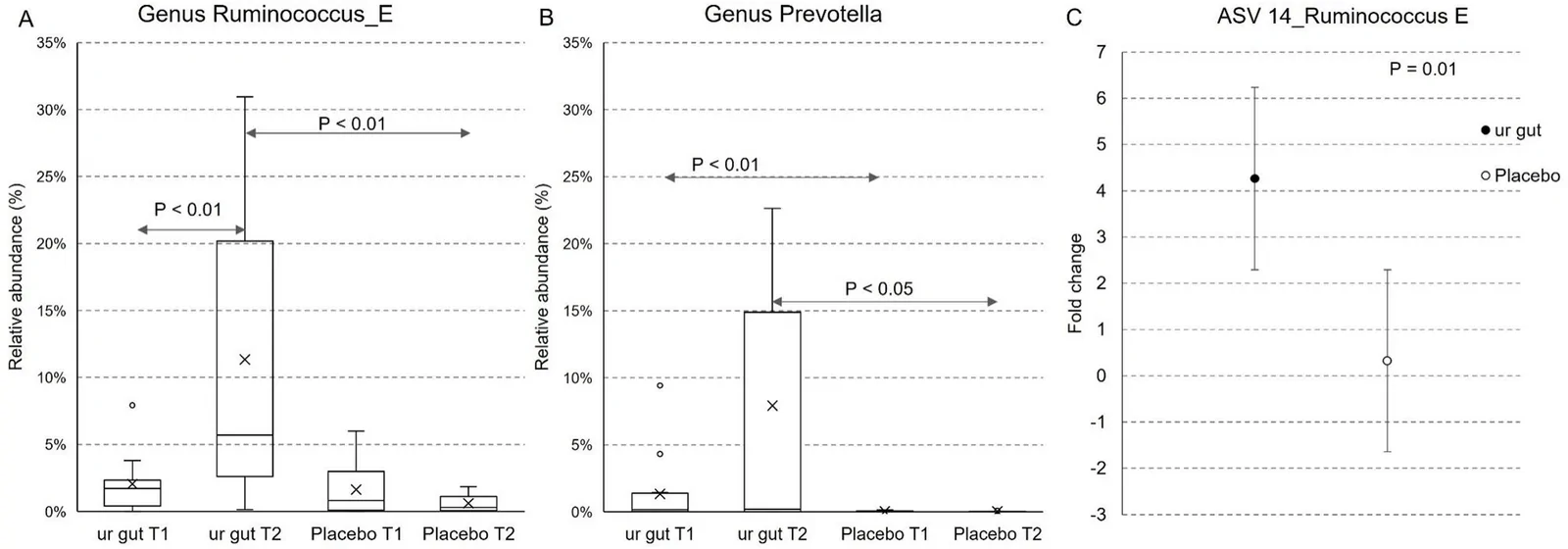
Changes in gut microbes. **A** The relative abundance of genus *Ruminococcus E* by group and timepoint. **B** The relative abundance of genus *Prevotella* by group and timepoint. **C** Fold change of *ASV 14 Ruminococcus E* within each group. Abbreviations: ASV, Amplicon Sequencing Variant; DASS21, Depression Anxiety Stress Scales; DistLM, Distance-based linear models; FDR, false discovery rate; FODMAP, fermentable oligosaccharides, disaccharides, monosaccharides, and polyols; GI, gastrointestinal; HAMS, high amylose resistant starch; hs-CRP, high-sensitivity C-reactive protein; IBS, irritable bowel syndrome; IBS-SSS, IBS-Symptom Severity Scale; Interleukin, IL; RS, resistant starch; SCFA, short chain fatty acid; TNF-α, Tumour Necrosis Factor alpha; QOL, quality of life; VSI, Visceral Sensitivity Index

The genus of *Ruminococcus E* increased to a relative abundance of 11.33% at T2 with a fold change (fc) of 5.5 (*P <* 0.01) in the ur gut^®^ group, where 92.3% (*n =* 12) of participants recorded an increase (Fig. 3A, Supplementary Table 12). This relative abundance was greater than that of the placebo group at T2 (*P <* 0.001, FDR *P =* 0.01) (Supplementary Table 12). Similarly, ur gut^®^ enriched *Prevotella*, fc = 5.9 (*P <* 0.05), which was more abundant at T2 compared to the placebo where the relative abundance of *Prevotella* remained unchanged (Fig. 3B, Supplementary Table 12). Among all classified genera, a total of four genera changed within the ur gut^®^ group, including *Ruminococcus E* (increased), *Anaerobutyricum* (decreased) and *Catenibacterium* (decreased), with none occurring in the placebo group (Supplementary Table 13, also showing between-group difference at T1 and T2). At ASV level within the ur gut^®^ group, a marginal effect was found in ASV 14 *Ruminococcus E*, 1.6% (T1) vs. 6.7% (T2), fc = 4.3 (*P =* 0.010, FDR.*P >* 0.05) (Fig. 3C, Supplementary Table 14). Specific ASV observations are provided in Supplementary Table 14.

### Gut microbiota- gut diversity

Regarding gut diversity indices, the placebo group was observed to have a lower diversity at baseline than the ur gut^®^ group, including Chao1, Shannon index and Fisher index (Supplementary Table 15). No interaction effects were noted after the intervention, where the indices remained stable in the ur gut^®^ group at T2 (Supplementary Table 15).

### Gut microbiota - distance-based linear models

DistLM analysis showed that eight variables individually explained a significant proportion of the variability of gut microbial composition at the three taxonomic levels of gut microbiota (phylum, genus and ASV) (Table 3). The Epworth Sleepiness Scale [54], indicating the daytime sleepiness contributed at all three taxonomic levels, together with the Pittsburgh Sleep Quality Index [50] at two levels, suggesting the influence of sleep on the gut microbiota composition (Table 3). At phylum level, daily dairy intake and number of daily bowel movement accounted for 13.3% and 6.7% (*P <* 0.05), respectively. Age and daily intake of resistant starch were found to account for 5.5% and 7.4% (*P <* 0.01), respectively, of the total gut microbial variation at genus level (Table 3). At ASV level, starch intake and IBS-SSS explained 5.6% (*P <* 0.05) and 6.2% (*P <* 0.01), respectively (Table 3). 

**Table 3:** Summary of variables explaining a significant individual proportion (%) of total gut microbial variation at three taxonomic levels at T2

Significant Variables	Individual Proportion (%) explained the total microbial composition variation in		
	Phylum level	Genus level	ASV level
ESS	9.8%	**7.7%**	5.4%
PSQI	**9.8%**		5.4%
Dairy, serve/d	13.3%		
# of Bowel Movement	6.7%		
Resistant starch, g/d		**7.4%**	
Age		5.5%	
Starch, g/d			5.6%
IBS-SSS			**6.2%**

The data was derived from 25 participants at T2. Only significant proportion was listed in the table (*P <* 0.05), and figures in bold indicate the variable’s *P <* 0.01. Acronym: ESS: The Epworth Sleepiness Scale; PSQI: The Pittsburgh Sleep Quality Index; # of bowel movement: mean of the number of bowel movements of the last three days which covered the dietary records period; IBS-SSS: Irritable Bowel Syndrome-Symptom Severity Scale. The sequential test variable order for each level is provided in supplemental Tables 16–18

## Discussion

This randomised trial is the first study to comprehensively explore tolerability of a mixed fermentable dietary fibre powder (ur gut^®^), and its effects on gut microbiota, sleep, mental health and QOL in IBS patients who are following a low FODMAP diet. The clinical relevance of the intervention lies not in increasing fermentable fibre intake per se, but in demonstrating that ur gut^®^ can be incorporated into a low-FODMAP dietary pattern without worsening IBS symptoms. The study outcomes showed that ur gut^®^ supplementation benefited overall gut microbial composition, selectively promoted gut microbes, and improved GI-specific anxiety and QOL (sexual subscale), without negative impacts on participants’ habitual sleep throughout the study. The presence of fibre is crucial to protect the intestinal barrier integrity and to balance microbial metabolic networks, whereby fibre-supplemented/fibre-rich diets can maintain the balance between mucin-degrading microbes and fibre-degrading microbes [65].

The demonstrated effect of ur gut^®^ is due to the unique blend of HAMS and psyllium. Rat studies have shown that the fermentation was shifted further distally when HAMS was mixed with psyllium [42]. Rats fed with HAMS+psyllium demonstrated a higher butyrate concentration, which was maintained throughout the colon, compared to rats fed HAMS only. If rats were fed low amylose maize starch (no RS)+psyllium, butyrate levels were lower in caecum, distal colon and faeces compared to HAMS+psyllium [42]. Moreover, rats fed HAMS+psyllium showed a 10-fold increase in caecal starch excretion and lower estimated microbial starch degradation in the large bowel (63% vs. 96% for HAMS alone), suggesting delayed fermentation and increased starch delivery to the distal colon [42]. Therefore, the unique fibre mix in ur gut^®^ is believed to slow proximal fermentability, where psyllium, with its strong gel-forming and water-holding capacity [66] can positively trap RS granules as a starch carrier to escape proximal fermentation. It prevents or slows proximal colonic fermentation, preventing or reducing gas production and related symptom-causing responses, and delivering RS further distally in the colon, where it is typically better tolerated. Previous research has shown the poor tolerability of RS alone in IBS sufferers [38], but our study has shown that the combination with psyllium husk powder can be well tolerated, which is fundamental to participants’ willingness to adhere to the intervention.

Gut microbes are highly dependent on their substrate preference [67], and as such, we found bacteria were selectively promoted in response to ur gut^®^. Our results showed that ur gut^®^ consistently enriched the genus *Ruminococcus E* and ASVs within this genus across participants. Compared to the negative change seen within the placebo group of that genus, this suggests ur gut^®^ provided a substrate for *Ruminococcus E.* As a primary RS degrader, *Ruminococcus.bromii*, a species within the genus *Ruminococcus*, can cross-feed other butyrogenic bacteria by increasing the concentration of acetate, which involves the carbohydrate-fuelled acetyl-CoA pathway - the primary pathway of butyrate generation in the colon [68, 69].Our results also showed that ur gut^®^ enriched the genus *Prevotalla*, which in turn can reflect long-term dietary patterns, where individuals with a diet based on carbohydrates and low in animal products feature an *Prevotella* enterotype [70]. In comparison to healthy subjects where the *Prevotella* enterotype is more prevalent, IBS patients commonly possess the *Bacteroides* enterotype, and symptom severity, as assessed by IBS-SSS, has been found to worsen as the prevalence of the *Prevotella* enterotype decreases [71]. Other studies have demonstrated a positive correlation between *Prevotella copri* and a fibre-rich diet [72], where those with non-Western lifestyles (including dietary factors) showed a higher prevalence of *Prevotella copri* compared to those with Western lifestyles (95% vs. 30%) [73].

The improvement in participants’ mental health (GI-specific anxiety assessed by VSI) and QOL (the sexual subscale of IBS-QOL) may be related to gut microbial alteration in relation to fibre supplementation. This study showed a greater abundance of *Alistipes* and *Coprococcus*, to be associated with more severe anxiety (VSI score). Moreover, *Alistipes* and *Coprococcus* had a fc of -1.2 and − 1.3, respectively, in the ur gut^®^ group at T2, yet participants had a significant reduction of VSI. Other studies have reported higher *Alistipes* abundance in IBS patients with co-morbidity of Myalgic Encephalomyelitis/Chronic Fatigue Syndrome [74], and in patients with major depressive disorder compared to healthy controls [75]; however, dietary analysis was not available for these studies. An elevated level of *Alistipes* was also exhibited in those consuming an animal-based diet both in the short term (5 days) [76] and longer term [77], likely related to a lower fibre intake, whereas a diet based on inulin-rich vegetables lowered *Alistipes* abundance in a healthy population [78]. Previous research has reported increased *Coprococcus_2* in people with small intestinal bacterial overgrowth and IBS-like symptoms compared with healthy controls [79], whereas reduced *Coprococcus has been observed* in those with depression [80]. This discrepancy suggest *Coprococcus* may be disease specific or context-dependent, differing between GI-related anxiety and depressive symptoms. In practical terms, GI-specific anxiety is the most influential factor affecting IBS-specific QOL, whereby psychological distress is also associated with overall QOL [75]. A 2–3 year follow-up study in females with IBS suggested that a high-fibre diet was associated with a significant decrease in anxiety and depression [81]. Regarding fibre interventions, its effects on mental health remain unclear, few recent IBS-related studies have included mental health measurements [77]. Although some prebiotic fibres have demonstrated potential to improve mood, they may be poorly tolerated in individuals with IBS as they fall within the FODMAP category [82]. Research has shown the microbiota-derived SCFA to directly or indirectly modulate signals between gut and brain, affecting brain and behaviour such as stress response, mood and appetite regulation [29, 83]. Accordingly, our outcomes suggest that ur gut^®^ may be a promising fibre supplement for mental health benefits and future studies are required to confirm its long-term effects.

No between-group changes were observed in SCFA excretion or concentration, which may be explained by both SCFA production and colonic absorption. Notably, > 95% of SCFA are absorbed by colonocytes, and this absorption in the human colon creates a negative concentration gradient throughout the colon [23]. Besides, baseline microbial composition plays a dominant role in shaping an individual’s response to dietary interventions [84]. In the post hoc analysis, both ur gut^®^_LOW_and placebo _LOW_ subgroups (baseline faecal output < 150 g/d) demonstrated increased faecal weight at T2, however, only the ur gut^®^ subgroup showed increases in butyrate concentration and excretion, suggesting that HAMS enhanced microbial fermentation while psyllium husk primarily contributed to stool bulking [34]. This provides preliminary evidence that IBS individuals with suboptimal faecal output (< 150 g/d) may be more responsive to ur gut^®^, where it can increase stool bulk and fermentation activity. However, it requires future studies to confirm these findings and to determine whether responses differ among IBS subtypes, given the exploratory nature of this subgroup post hoc analysis.

When ur gut^®^ was introduced to participants’ background low FODMAP diet, the severity of symptoms and sleep throughout the study did not change, nor did DQI and FODMAP intakes. ur gut^®^ was well tolerated and accepted by participants, resulting in their continued compliance. Notably, ur gut^®^ was introduced over 11 days, increasing from 5 g/d to 40 g/d, taken as a 20 g dose twice a days over the next 10 days, which is aligned with current recommendations of an adaptation period for most fibre supplements [85]. Additionally, the effect of ur gut^®^ on reducing energy intake was aligned with participant anecdotal feedback of ‘feeling fuller after taking the supplement’ on the final clinic visit. This was not an anticipated outcome of this study but is an important consideration for those living with overweight/obesity. Research has indicated that an intake of 8 g/d viscous fibre for 8 weeks can lead to a significant reduction in body weight for the general population and those with diabetes/obesity/overweight [86], although a longer supplementation period with isolated soluble fibre may be needed for those with overweight/obesity [87].

### Limitations

Limitations include a small sample size, dominance of female participants and differing IBS subtypes. IBS is more prevalent in women than men with females accounting for approximately 60–70% of diagnosed cases in Western populations. Gender differences are also evident across IBS subtypes, with IBS-C predominating in women, while IBS-D is more commonly reported in men. Several results showed marginal significances (namely raw *P ≤* 0.05, but FDR-adjusted *P >* 0.05), which could be attributed to our relatively small sample size, especially in gut microbial analysis. Gut transit time and faecal pH were not assessed in the present study, which should be incorporated in future research to strengthen methodological completeness. Further research with larger sample sizes is required to identify the long-term effects or ur gut^®^ fibre supplementation, with alternative dosing strategies, on gut health and metabolic health in broader IBS populations as well as its potential application in other GI disorders or diseases.

## Conclusion

Compared with placebo, supplementation with a specific combination of fibres increased total dietary fibre intake without worsening IBS symptoms in individuals following a low-FODMAP diet. Adding ur gut^®^ to this group’s habitual low FODMAP diet improved GI-specific anxiety and benefited gut microbial composition, without negatively impacting participants symptoms severity, sleep and dietary quality and patterns. Given the small sample size and short duration of the present study, the observed mental health findings should be interpreted with caution, and future research in needed to determine its long-term effects in a larger population. These findings provide preliminary evidence that ur gut^®^ may represent a feasible dietary strategy to support gut health without exacerbating symptoms in individuals with IBS.

## Supplementary Information

Supplementary Material 1.

## Data Availability

The data that support the findings of this study are not publicly available but are available from the corresponding author on reasonable request.

## Abbreviations

ASV: Amplicon Sequencing Variant
DASS21: Depression Anxiety Stress Scales
DistLM: Distance-based linear models
FDR: False discovery rate
FODMAP: Fermentable oligosaccharides, disaccharides, monosaccharides, and polyols
GI: Gastrointestinal
HAMS: High amylose resistant starch
hs-CRP: High-sensitivity C-reactive protein
IBS: Irritable bowel syndrome
IBS-SSS: IBS-Symptom Severity Scale
IL: Interleukin
RS: Resistant starch
SCFA: Short chain fatty acid
TNF-α: Tumour Necrosis Factor alpha
QOL: Quality of life
VSI: Visceral Sensitivity Index
