# Supplementary material for: Dietary fibre blend improves gut health in patients with irritable bowel syndrome following a low FODMAP diet: a randomised, double-blind control trial
Source: Eur J Nutr. 2026 Sep 24;65(7):263. doi: 10.1007/s00394-026-04113-5 (PMC13612624; doi:10.1007/s00394-026-04113-5)
Supplement: Supplementary file 1 [file 394_2026_4113_MOESM1_ESM.docx]

**Title:**

Dietary fibre blend improves gut health in patients with Irritable Bowel Syndrome following a low FODMAP diet: a randomised, double-blind control trial

**Authors' institutions**

Ran Yan [^1^](https://tas.currinda.com/event/2247/submit/464/16889/preview#affiliation_23824)^,2^, Amanda Devine [^1^](https://tas.currinda.com/event/2247/submit/464/16889/preview#affiliation_23824)^,2^, Johnny Lo [^3^](https://tas.currinda.com/event/2247/submit/464/16889/preview#affiliation_23828),  Evania Marlow [^1^](https://tas.currinda.com/event/2247/submit/464/16889/preview#affiliation_23824)^,2,^[^4^](https://tas.currinda.com/event/2247/submit/464/16889/preview#affiliation_23829), Ian C Dunican [^1^](https://tas.currinda.com/event/2247/submit/464/16889/preview#affiliation_23824)^,5^, Kanita Kunaratnam [^1,2^](https://tas.currinda.com/event/2247/submit/464/16889/preview#affiliation_23829), Lesley Andrew ^6^, Claus T Christophersen [^1^](https://tas.currinda.com/event/2247/submit/464/16889/preview#affiliation_23824)^,2^

**Authors' institutions**

1. School of Medical and Health Sciences, Edith Cowan University, Joondalup, WA, Australia
2. Nutrition and Health Innovation Research Institute, Edith Cowan University, Joondalup, WA, Australia
3. School of Science, Edith Cowan University, Joondalup, WA, Australia
4. Joondalup Health Campus, Joondalup, WA, Australia
5. Centre for Sleep Science, School of Human Sciences, The University of Western Australia, Crawley, Western Australia, Australia.
6. School of Nursing and Midwifery, Edith Cowan University, Joondalup, WA, Australia

**Corresponding Author:**

Ran YAN

School of Medical and Health Sciences, Edith Cowan University

270 Joondalup Drive, Joondalup, WA

^E:^ [^r.yan@ecu.edu.au^](mailto:r.yan@ecu.edu.au)

# Supplementary Methods

## Patient and Public Involvement

Patients with IBS were involved in the recruitment to the study, where a rolling recruitment occurred from July 2020– July 2021 in Perth, Western Australia.

## Inclusion and exclusion criteria

Those who met the inclusion criteria were recruited into the study. These included: men and women between 18 – 65 years old with a clinical diagnosis of IBS, using the Rome IV edition diagnostic criteria ^2^, diagnosed by a gastroenterologist or other medical professional; those who had been on a low FODMAP diet for at least one month prior to the intervention and had stable symptoms (self-reported); those who were available to attend local clinic visits and were willing to consume the ur gut® supplement or matched placebo; and those who had a BMI between 18.5 – 40 kg/m^2^.

Participants were excluded if they: were current smokers; were pregnant or planning to become pregnant; had a known diagnosis of other gastrointestinal illness (e.g., Inflammatory Bowel Disease, malabsorption of any macronutrients, bowel resection, coeliac disease); had undergone previous abdominal or gastrointestinal surgeries, had severe mental health and sleep-related conditions (e.g., insomnia); had renal or hepatic diseases, and major medical illness; currently used pharmaceutical agents that could modify or treat IBS (e.g., probiotics, antibiotics, eluxadoline, lubiprostone and linaclotide) or sleep conditions; followed other restrictive dietary patterns or therapies (e.g., low-carbohydrate, or high-protein diets, keto/Paleo-diet); took any prebiotics; had any other diseases, conditions or habits that may interfere with completion of the study.

The reason of inclusion of all IBS subtypes was the ur gut® fibre, where psyllium was the one of the ingredients. Psyllium has been identified with its bidirectional effects – normalising the stool consistency and frequency: softening hard stool or firming loose/liquid stool regardless of IBS subtypes ^3^. As this is the first study of ur gut® fibre, and it would be ideal to identify any potential IBS population that could benefits from the fibre.

In response to the COVID 19 pandemic, which caused difficulty in recruitment, the eligibility criteria relevant to probiotic and antibiotic use was relaxed to include those who were willing to stop taking current probiotics/antibiotics for at least 3 weeks prior to study commencement and cease using them during the study. Accordingly, these eligible participants completed a 3-week wash-out prior to the starting date.

## Diet Quality Index

Aussie-DQI (a diet quality index for Australian population) ^4^ was used for assessing general diet quality, where it has been listed as one of the preferred DQI used in Australian adults according to a systematic review in 2020 ^5^. Aussie-DQI is comprised of 11 components, each scored out of ten points (ten indicating the guideline was sufficiently met), with one component having four sub-components and each was scored out of five points. The detailed components and scoring instruction were adapted from *Table 1* of the original Aussie DQI validation paper ^4^, thereby making the total Aussie-DQI score within a range of 0 to 120. Higher Aussie-DQI scores indicate a better diet quality with desirable intake of nutrients, a greater adherence to the Australian Dietary Guidelines (ADG) ^6^, and inversely, is associated with risk of cancer mortality ^4^.

In 2017, ADG revised the Suggested Dietary Target to 2,000 mg/d for adults and ‘not determined’ for sodium ^7^. After consultation with authors of the Aussie-DQI, we agreed to adjust the cut-off of sodium to align with the latest recommendation of 2,000 mg/d. This also suggests the results by Zarrin et al ^7^ are slightly outdated. Despite this, it is mostly correct and provides a valuable baseline reference for the current study.

## Education of consumption and calculation of compliance

Participants were educated about the best procedure for consuming the supplement. The intervention period - a total of three weeks, consisted of two phases: a ramping-up phase of 11 days where fibre dose increased from 5 g/d to 40 g/d, followed by a full-dose phase of 10 days at 40 g/d. The introductory design aimed to accustom participants’ GI tract to the extra fibre intake where their habitual diet provided a certain amount of background fibre intake, and participants were required to maintain their diet throughout the 4-week study period.

Participants were recommended to take the supplement immediately after mixing it thoroughly with at least 320 ml water or a beverage at room temperature after stirring thoroughly for 20 seconds (before the fibre settles to the bottom). Moreover, participants were instructed to not heat/cook/bake the supplement, or add the mixture to hot drinks above 60 °C (140 °F) to avoid altering fibre content. Participants were also instructed to record the time of consumption via the online daily checklist. If a sachet was missed, participants were instructed to keep consuming sachets as scheduled, and bring back empty packages and missed sachets.

Both sachet packages and checklist records were used to calculate the compliance. Consumption of greater than 80% of the whole amount of the fibre supplement over the 3-week intervention was considered compliant with the study protocols. Therefore, participants who did not meet the criterion were regarded as non-complaint and were excluded from the per-protocol analysis.

## IBS symptoms collection

The record of IBS symptoms was conducted daily via the online daily checklist and at the two timepoints via validated questionnaires, the Gastrointestinal Symptom Rating Scale for IBS, and the Irritable Bowel Syndrome-Symptom Severity Scale (IBS-SSS).

Enrolled participants were required to have had globally stable symptoms, as inclusion criteria outlined. The symptomatic improvement of a low FODMAP diet has been evidenced by existing research, whereas this study aimed to determine whether the fibre intervention is tolerable in people with IBS, supporting gut health without exacerbating gastrointestinal symptoms. Therefore, the requirement for relatively stable baseline symptoms enabled clearer identification of potential flare-ups during the fibre intervention. This approach reduced the risk of incorrectly attributing symptom changes to the intervention when they may have been caused by underlying dietary or lifestyle factors.

## Blood sample collection

Venous blood samples were collected at both first and final clinic visits taken by a trained phlebotomist at Edith Cowan University (ECU). Participants were required to fast overnight from 10 pm the night before their morning appointment. Approximately 15 ml of blood was collected in three kinds of vacuum blood collection tubes, 5 ml premium VACUETTE® tube serum gel gold cap, 4 ml premium VACUETTE® tube lithium heparin, and 4 ml premium VACUETTE® tube K3 EDTA, using ECU Standard Operating Procedures for Human Phlebotomy. Following Standard Operating Procedures for Human Blood Centrifuging and Aliquoting, the collected samples were stood to clot for 30 minutes and centrifuged before separating plasma and serum, and then frozen at −80 °C after being aliquoted into 2 ml vials.

Blood sample analysis

Analysis of cytokines (High-sensitive Interleukin-6 (IL-6), IL-1β, tumour necrosis factor-α (TNF-α), and high-sensitive C reaction protein (hs-CRP) was assessed in accordance with the protocols from the National Association of Testing Laboratories by SA pathology laboratory in Adelaide, South Australia. Their assay cytokine test has received National Association of Testing Authorities, Australia (NATA) accreditation. Cytokines, high-sensitive IL-6, IL-1β, TNF- α, were measured in serum using BD™ Cytometric Bead Array (CBA) Kits (BD Biosciences, Sans Jose, CA, USA) with the addition of the BD™ CBA Enhanced Sensitivity Flex Set, a bead-based immunoassay, being capable to quantitate soluble analytes present in very low concentrations. Following the manufactures’ assay instructions, the BD™ CBA kit was used in conjunction with a BD CBA Human Enhanced Sensitivity Master Buffer Kit, a flow cytometer on BD FACSCanto, and FCAP Array™ Software for data analysis (BD Biosciences, Sans Jose, CA, USA). For the measurement of hs-CRP in serum, particle enhanced immunoturbidimetric assay (Roche Diagnostics, Indianapolis, IN, USA) was used following manufacturers instruction. The lower detection limits of the all tests summarised as followed: IL-1β: 0.048 pg/mL; IL-6: 0.068 pg/mL; TNF- α: 0.067 pg/mL; hs-CRP: 0.15 mg/L.

## 24-hour stool collection

Given the real‑life, home‑based study design, participants were provided with a stool collection kit, including a portable cooler bag, frozen icepacks and an instruction sheet. Participants were instructed to collect the whole stool sample of each bowel movement within the 24-h period, and stored faecal samples in the provided esky with ice packs. Upon receipt, stool samples were immediately weighed and stored at −80 °C, according to ECU Standard Operation Procedure for stool collection. To generate 24-hour faecal output, all weights of stool samples were summed and the total weight of collecting bags and cable ties was subtracted.

## Faecal SCFA analysis

Faecal SCFA analysis was undertaken at ECU. The concentration of SCFA was determined by gas chromatography ^8^. In brief, an acidified aqueous methanol solution was used to extract SCFA from faecal samples, followed by separating of SCFA by gas chromatography with a fatty acid column (Zebron ZB-FFAP 30m x 0.53mm x 1m μm supplied by PhenomeneX). The SCFA concentrations were determined via internal standards. The SCFA outcome measures included six acids: acetic acid, propionic acid, isobutyric acid, butyric acid, isovaleric acid and valeric acid. These six acids added together to calculate the total SCFA concentration. The acid concentration was measured in μmol/g of wet faeces. The excretion amount of each SCFA type and all SCFA was calculated by multiplying the respective concentration by the participant’s total 24-hour stool weight (gram), and the unit was mmol per 24 hours.

## Gut microbiota analysis

Microbial analyses were performed at the WA Human Microbiome Collaboration Centre, Curtin University, Western Australia. The analysis of the gut microbiota was similar to the approach in the study of Jones et al ^9^. In summary, DNA was extracted using the QIAamp PowerFecal Pro DNA kit (Qiagen) using Qiacube extraction platform following PCR inhibition assessment of each DNA extract. PCR-free ligation protocol was thereafter deployed for the process of library building. Microbiome signatures were generated using the Illumina MiSeq platform using uniquely barcoded 16S rRNA gene primers (515-806 (V4)) for quality control, mock communities, extraction controls and non-template control samples were included in the sequence analysis. Sequence read quality was initially assessed with FastQC before demultiplexing and pre-processing by GHAPv2, an in-house tool. Cutadapt ^10^ was used for removal of all non-biological sequences. DADA2 ^11^ was then used for quality filtering, error correction, amplicon sequence variants (ASVs) picking. A trained naïve bayes classifier then assigns ASVs to genus/species against a curated database of microbial reference sequences, Genome Taxonomy Database ^12^. If the microbes were not able to be identified at genus or species level, the Basic Local Alignment Search Tool (BLAST) of National Center for Biotechnology Information (NCBI) will be used to identify via the comparison the nucleotide sequences of the specific ASV to the sequence databases. The sequence data yielded a total of 3031 ASVs which were further quality filtered according to the five principles below and in the order:

1) Each ASV had greater than or equal to 100 reads in total (1304 ASVs included).
2) Each ASV was represented in more than 5% (n=3) of the total numbers of samples (1095 included)
3) The reads in the Non-Template Control counting for the ASV reads were below 1% (1058 included)
4) The reads in the Extraction Control counting for the ASV total read were below 1% (1054 included)
5) Any ASV not classified beyond Kingdom (with assigned phylum) was deleted.

This resulted in a total of 988 reads.

## Statistical analysis

For daily sleep measurements, those who had >50% complete data for each phase of the entire study, including Phase 1: a baseline week (7 days), Phase 2: dose ramping-up period (11 days), and Phase 3: full dose period (10 days) were included in the analysis.

The total 24-h stool weight (wet weight) was calculated and assessed to be above or below 150 g/d, a faecal index relevant to colon cancer risk ^13^. Anthropometric data, results of stool SCFA and blood biomarkers, dietary assessment data (FODMAP, nutrients and food groups), scores of all questionnaires, daily objective sleep data collected via Readiband, as well as scores of symptom severity collected from daily checklist were all exported to MS Excel ^14^ for storage and cleaning. Missing data and outliers were checked for scientific accuracy. The resulting spreadsheets were imported into SPSS v27.0 ^15^ for analysis.

All continuous outcome variables were examined for normality using the Shapiro-Wilk test. Descriptive statistics in the form of mean and standard deviation (Std. D) were used to describe numerical variables that were normally distributed, and an independent t-test was conducted to identify the difference between the placebo and intervention group at baseline. Median and interquartile range (IQR) were reported for non-normally distributed data, whereby a Mann-Whitney U test was used instead. For nominal variables, frequencies and proportions were reported; the distribution of categorical variables across each group was tested using the Pearson Chi-square test. False discovery rate (FDR) correction was performed on all raw P-values to minimise false positive results. When unadjusted (raw) P was ≤ 0.05 and FDR-adjusted P ≥ 0.05, it would be noted as marginal significance/ evidence suggesting a trend, whereas FDR-adjusted P ≤ 0.05 would be deemed significant, and with a symbol (or a superscript) marking the P value.

With regard to gut microbiota, at the levels of phylum, genus and ASV, the relative abundance data were square root transformed and a Bray-Curtis dissimilarity matrix was calculated, followed by the deployment of Principal Coordinates Analysis (PCoA) to visualise findings of the three taxonomic levels at T1 and T2. For the determination of significant associations at baseline and longitudinal dissimilarities between the two groups, PERMANOVA and dissimilarity percentage (SIMPER) were conducted. Distance-based linear models (DistLM) and distance-based redundancy analysis (dbRDA) were used to integrate gut microbial findings with all other relevant aspects, such as: dietary intake, sleep, mental health and quality of life, blood and faecal biomarkers at each of the three levels contributing to 80% of participants' gut microbial composition variations, in order to account for the variability of the gut microbial composition at each level. For the DistLM analysis of the T2 result, one participant was removed due to their incomplete questionnaire. A stepwise selection procedure was applied in the DistLM analysis, with R² used as the variable selection criterion. The dbRDA ordination was utilised to visualise variables, which contributed to 80% of the variations of gut microbial composition of participants.

The Shannon index, Chao1 index and Fisher index were three indices used to indicate alpha microbial diversity using rarefied data at both timepoints. These indices, and the observed species, were exported to SPSS v27.0 ^15^ for LMM analysis to identify any between-group and within-group changes with BMI, and were age adjusted in the model. The significance level was set at P ≤ 0.05.

# SUPPLEMENTARY FIGURES

Supplementary Figure 1. CONSORT diagram of the study.

A1.Individual energy intake in the ur gut group

|  | UG3 | UG5 | UG6 | UG8 | UG14 | UG17 | UG18 | UG19 | UG20 | UG21 | UG23 | UG26 | UG28 |
| --- | --- | --- | --- | --- | --- | --- | --- | --- | --- | --- | --- | --- | --- |
| T1 Energy_kJ/d | 8524.01 | 7686.9 | 10335.7 | 5296.8 | 9604.46 | 8590.73 | 6913.4 | 11095.2 | 12327.7 | 7663.26 | 5329.55 | 7355.42 | 6540.18 |
| T2 Energy_kJ/d | 7293.11 | 5303.64 | 10610.8 | 5570.28 | 6593.9 | 5417.9 | 6293.37 | 10570.4 | 9843.11 | 6483.6 | 5958.82 | 7588.08 | 5881.82 |

A2.Individual energy intake in the plaecbo group

|  | PG1 | PG2 | PG4 | PG7 | PG11 | PG12 | PG13 | PG16 | PG24 | PG25 | PG27 | PG29 | PG30 |
| --- | --- | --- | --- | --- | --- | --- | --- | --- | --- | --- | --- | --- | --- |
| T1 Energy_kJ/d | 12258.5 | 6450.99 | 11642.2 | 6004.24 | 8658.62 | 9308.36 | 6860.48 | 5651.57 | 4947.97 | 8014.52 | 5992.36 | 5782.56 | 8289.73 |
| T2 Energy_kJ/d | 10265.7 | 6124.88 | 10247.9 | 5850.86 | 10252.8 | 10373.5 | 4559.94 | 7500.81 | 6642.5 | 20670.5 | 7160.81 | 7880.45 | 10915.9 |

B1. Individual protein intake in the ur gut group

|  | UG3 | UG5 | UG6 | UG8 | UG14 | UG17 | UG18 | UG19 | UG20 | UG21 | UG23 | UG26 | UG28 |
| --- | --- | --- | --- | --- | --- | --- | --- | --- | --- | --- | --- | --- | --- |
| T1 Protein_g/d | 98.4335 | 61.4719 | 109.956 | 74.3781 | 98.0299 | 126.899 | 70.6463 | 95.7975 | 195.062 | 94.7305 | 86.8821 | 93.1614 | 70.0528 |
| T2 Protein_g/d | 64.1951 | 60.3138 | 114.776 | 65.1838 | 80.797 | 83.3163 | 55.303 | 82.9952 | 141.749 | 78.7074 | 80.6452 | 70.1436 | 67.5598 |

B2. Individual protein intake in the placebo group

|  | PG1 | PG2 | PG4 | PG7 | PG11 | PG12 | PG13 | PG16 | PG24 | PG25 | PG27 | PG29 | PG30 |
| --- | --- | --- | --- | --- | --- | --- | --- | --- | --- | --- | --- | --- | --- |
| T1 Protein_g/d | 157.435 | 80.8718 | 127.882 | 26.8324 | 100.87 | 93.4204 | 92.9741 | 48.5228 | 85.4422 | 51.6489 | 35.5306 | 55.9524 | 85.7738 |
| T2 Protein_g/d | 194.981 | 56.9382 | 141.626 | 36.2389 | 98.4503 | 144.704 | 45.3026 | 79.2804 | 85.5216 | 90.4921 | 61.8102 | 88.437 | 72.9057 |

C2. Individual fibre intake in the placebo group

|  | PG1 | PG2 | PG4 | PG7 | PG11 | PG12 | PG13 | PG16 | PG24 | PG25 | PG27 | PG29 | PG30 |
| --- | --- | --- | --- | --- | --- | --- | --- | --- | --- | --- | --- | --- | --- |
| T1 Dietary fibre g/d | 29.7705 | 15.9989 | 21.4161 | 25.1592 | 23.1749 | 28.2094 | 10.1522 | 22.1412 | 20.8374 | 10.4103 | 15.6894 | 16.5998 | 35.9908 |
| T2 Dietary fibre g/d | 35.276 | 17.4561 | 20.2378 | 35.2285 | 19.7 | 47.1197 | 17.6524 | 31.5371 | 15.8294 | 34.4228 | 24.1951 | 29.5195 | 48.6918 |

C1. Individual fibre intake in the ur gut group

|  | UG3 | UG5 | UG6 | UG8 | UG14 | UG17 | UG18 | UG19 | UG20 | UG21 | UG23 | UG26 | UG28 |
| --- | --- | --- | --- | --- | --- | --- | --- | --- | --- | --- | --- | --- | --- |
| T1 Dietary fibre g/d | 18.9085 | 15.2866 | 18.4605 | 12.3104 | 23.0324 | 24.1593 | 26.2259 | 17.4708 | 34.4781 | 18.3735 | 12.7329 | 9.73186 | 31.9138 |
| T2 Dietary fibre g/d | 28.947 | 28.207 | 32.801 | 26.0814 | 29.3351 | 32.7561 | 32.8456 | 34.6068 | 48.0918 | 34.7918 | 27.3737 | 27.6785 | 47.493 |

D2. Individual resistant starch intake in the placebo group

|  | PG1 | PG2 | PG4 | PG7 | PG11 | PG12 | PG13 | PG16 | PG24 | PG25 | PG27 | PG29 | PG30 |
| --- | --- | --- | --- | --- | --- | --- | --- | --- | --- | --- | --- | --- | --- |
| T1 Dietary fibre g/d | 1 | 1.5107 | 3.31917 | 4.28853 | 2.7112 | 3.30966 | 3.55005 | 2.36814 | 0.643749 | 1.51259 | 0.858491 | 1.32141 | 1.73424 |
| T2 Dietary fibre g/d |  | 1.72619 | 4.14488 | 3.0245 | 3.4 | 3.98127 | 1.90407 | 1.90705 | 1.97604 | 1.98448 | 1.82569 | 1.77307 | 1.90634 |

D1. Individual resistant starch intake in the ur gut group

|  | UG3 | UG5 | UG6 | UG8 | UG14 | UG17 | UG18 | UG19 | UG20 | UG21 | UG23 | UG26 | UG28 |
| --- | --- | --- | --- | --- | --- | --- | --- | --- | --- | --- | --- | --- | --- |
| T1 Dietary fibre g/d | 2.91516 | 1.43803 | 2.22074 | 0.572083 | 2.03785 | 2.54916 | 5.08395 | 2.30653 | 3.28064 | 0.99595 | 0.265267 | 1.70011 | 0.150038 |
| T2 Dietary fibre g/d | 10.0399 | 9.55834 | 10.505 | 8.09512 | 9.48652 | 10.4576 | 10.7234 | 9.7703 | 10.084 | 9.14757 | 9.12837 | 9.9349 | 7.06815 |

Supplementary Figure 2. Comparision of (A) energy intake, (B) protein intake, (C) fibre intake and (D) resistant starch intake for individual participants in both groups between Timpoint 1 and Timepooint 2.

Supplementary Figure 3. Comparison of sleep measures by group and phase.

The horizontal lines are sourced from National Sleep Foundation's sleep quality recommendations ^1^ **A.** Sleep Latency. if < 30 mins is indicative of good sleep quality. **B.** Time At Sleep Onset. **C.** Fragmentation Index; ≤ 1 is an indicator good sleep quality. **D.** Wake After Sleep Onset; < 20 mins is an indicator of good sleep quality; > 40 mins is indicative of poor sleep quality. **E.** Sleep Duration, 7 ~ 9 hours is recommended. **F.** Time At Wake; **G.** Time in Bed; **H**. Sleep Efficiency; ≥ 85% was an indicator of good sleep quality.

Supplementary Figure 4. PCoA plots showing the ASV level of gut microbiota by group and timepoint. **A.** The PCoA plot showing ASV-level gut microbiota of the two groups at T1. **B.** The PCoA plot showing ASV-level gut microbiota of the two groups at T2. The green triangle represents the ur gut® group, and the red circle represents the placebo group. ASV, Amplicon sequence variant.

# Supplementary Results

## Participants

Patient and Public Involvement

A rolling recruitment occurred for this research from July 2020 July – July 2021 in Perth, Western Australia. Overall, 30 participants enrolled after signing the online consent form; however, one female participant withdrew during the baseline observation due to pregnancy, and three participants did not complete the study (Supplementary Figure 1). In the placebo group, one participant was lost to follow-up and another self-reported feeling unwell and withdrew. One female in the ur gut® group reported nausea and withdrew. Therefore, 26 participants completed the study and were included in the analysis (Supplementary Figure 1), which fulfilled the minimum required sample size of 24 participants. Out of all participants, 73% had a diagnosis of IBS beyond one year, whereas 35% had been on a low FODMAP diet for more than one year, and 77% were at the phase of a personalised low FODMAP diet (Table 1). On average, participants had a healthy weight, level of adiposity and blood pressure, and engaged in physical activity, whereby no significant change was noted in anthropometrics and physical activity (Supplementary Table 1).

## Faecal SCFA

Suggestive evidence was noted for a Group × Time interaction (P = 0.042, FDR-adjusted P > 0.05) between the two sub-groups in the ur gut® group, the ur gut® _HIGH_ (n = 8, T1 faecal output > 150 g/d) and the ur gut® _LOW_ (n = 5，T1 faecal output < 150 g/d), where the latter had an increase in the mean faecal output to 161.9 g/d at T2 (no change in the daily number of bowel movements, Supplementary Table 9). Individually, four of the five participants in the ur gut® _LOW_ subgroup had the faecal output above 150 g/d at T2, whilst only one third of participants had this response in the placebo _LOW_ subgroup. For SCFA, marginal evidence of interaction effects was noted in the excretion amount of total SCFA, acetate and butyrate, as well as butyrate concentration, where the ur gut® _LOW_ subgroup had significant increases (except for total SCFA) in comparison to its T1 level and the ur gut® _HIGH_ group did not significantly change (Supplementary Table 9). Within the placebo group, marginal evidence of interaction effects was observed for faecal output (P = 0.036, FDR-adjusted P > 0.05), where the Placebo _HIGH_ (n = 8, T1 faecal output > 150 g/d) had a significant reduction by -44.1% (P = 0.026) (Supplementary Table 10).

# Supplementary Tables

| Supplementary Table 1 Demographic information by group | | | |  |
| --- | --- | --- | --- | --- |
| Demographic information | Total, N=26, n (%) | ur gut® Group n=13, n (%) | Placebo Group  n=13, n (%) | P |
| Mean age, years, mean (Std. D) | 37 (13) | 38 (10) | 37 (15) | 0.194 |
| Gender |  |  |  | 1.000 |
| Male | 3 (12) | 2 (15) | 1 (8) |  |
| Female | 23 (88) | 11 (85) | 12 (8) |  |
| Ethnic Background |  |  |  | 1.000 |
| Caucasian (White) | 17 (65) | 8 (62) | 9 (69) |  |
| Other | 9 (35) | 5 (38) | 4 (31) |  |
| Highest Education Achieved |  |  |  | 0.522 |
| Secondary school, TAFE /Technical/Business college | 17 (65) | 10 (77) | 7 (54) |  |
| University, Tertiary education and higher | 9 (35) | 3 (23) | 6 (46) |  |
| Marital Status: |  |  |  | 0.709 |
| Never married /Single/Not married | 7 (27) | 3 (23) | 4 (31) |  |
| Married/De facto/Divorced /Widowed/  Separated married(in a registered marriage) | 19 (73) | 10 (77) | 9 (69) |  |
| Delivered Mode |  |  |  | 0.330 |
| Vaginal | 15 (58) | 9 (69) | 6 (55) |  |
| Caesarean section | 4 (15) | 1 (8) | 3 (27) |  |
| Unknown | 7 (27) | 3 (23) | 4 (36) |  |
| Smoking Status |  |  |  | 1.000 |
| Never | 19 (73) | 9 (69) | 10 (77) |  |
| Used to | 7 (27) | 4 (31) | 3 (23) |  |
| Alcohol consumption |  |  |  | 1.000 |
| Never | 3 (12) | 1 (8) | 2 (15) |  |
| Yes | 23 (88) | 12 (92) | 11 (85) |  |
| IBS subtype |  |  |  | 0.475 |
| Constipation | 5 (19) | 3 (23) | 2 (15) |  |
| Diarrhoea | 6 (23) | 4 (31) | 2 (15) |  |
| Mixed | 10 (38) | 3 (23) | 7 (54) |  |
| Undefined | 5 (19) | 3 (23) | 2 (15) |  |
| Low FODMAP diet phase |  |  |  | 1.000 |
| Restriction | 3 (12) | 1 (8) | 2 (15) |  |
| Reintroduction | 3 (12) | 2 (15) | 1 (8) |  |
| Personalisation | 20 (77) | 10 (77) | 10 (77) |  |
| Low FODMAP diet + other diary patterns |  |  |  | 0.520 |
| No other dietary patterns | 19 (73) | 11 (85) | 8 (62) |  |
| Gluten-free/Diary-free/ Both | 3 (12) | 1 (8) | 2 (15) |  |
| Vegetarian/ Vegan/ Flexitarian | 3 (12) | 1 (8) | 2 (15) |  |
| Both Gluten-free/Diary-free and Vegetarian /Vegan/Flexitarian | 1 (4) | 0 (0) | 1 (8) |  |
| Diagnosis of IBS |  |  |  | 0.349 |
| ≤ 1 year | 7 (27) | 3 (23) | 4 (31) |  |
| > 1 year | 19 (73) | 10 (77) | 9 (69) |  |
| diagnosed with IBS, years, median (IQR) | 5 (14) | 5 (11.5) | 7 (21.9) | 0.762 |
| Low FODMAP diet |  |  |  | 0.505 |
| ≤ 1 year | 17 (65) | 9 (69) | 8 (62) |  |
| > 1 year | 9 (35) | 4 (31) | 5 (38) |  |
| Following a low FODMAP diet, months, median (IQR) | 12 (21.8) | 12 (20) | 8 (55.5) | 0.840 |
| Note: Std. D: standard deviation; IQR: interquartile range; IBS: Irritable Bowel Syndrome; FODMAP: fermentable oligosaccharides, disaccharides, monosaccharides and polyols. Independent t-tests (for age) and Mann-Whitney U tests were conducted to identify the between-group difference at T1. P-values are unadjusted. | | | | |

Supplementary Table 2. Daily GI symptoms by group and phase

| Daily Measures | Group × Time Interaction P | ur gut® Group, n = 13  Mean (SE) | | | Placebo Group, n = 13  Mean (SE) | | | Between-group P |
| --- | --- | --- | --- | --- | --- | --- | --- | --- |
|  |  | Phase 1 | Phase 2 | Phase 3 | Phase 1 | Phase 2 | Phase 3 |  |
| IBS-SSS^d^ | 0.304 | 139.0 (20.1) | 161.8 (19.4) | 173 (19.5) | 186.0 (20.0) | 186.3 (19.3) | 181 (19.6) | 0.373 |
| Items on the daily checklist (individual symptoms) | | | | | | | | |
| Sleep quality (10 best) | 0.931 | 5.6 (0.4) | 5.8 (0.4) | 6.2 (0.4) | 5.7 (0.4) | 6.4 (0.4) | 6.3 (0.4) | 0.471 |
| Stress (10 worst) | 0.188 | 3.7 (0.7) | 3.6 (0.7) | 3.6 (0.7) | 3.9 (0.7) | 3.5 (0.7) | 3.1 (0.7) | 0.842 |
| Bloating | 0.482 | 3.8 (0.6) | 3.9 (0.6) | 3.9 (0.6) | 4.5 (0.6) | 4.0 (0.6) | 4.0 (0.6) | 0.786 |
| Gas | 0.245 | 3.7 (0.6) | 4.3 (0.5) | 3.9 (0.5) | 3.6 (0.6) | 3.4 (0.5) | 3.7 (0.5) | 0.566 |
| Stomach pain | 0.877 | 2.6 (0.5) | 2.5 (0.5) | 2.5 (0.5) | 3.3 (0.5) | 2.9 (0.5) | 3.1 (0.5) | 0.457 |
| Diarrhea | 0.329 | 2.0 (0.4) | 1.4 (0.4) | 1.5 (0.4) | 1.3 (0.4) | 1.1 (0.4) | 1.2 (0.4) | 0.478 |
| Burping | 0.357 | 1.7 (0.6) | 1.8 (0.6) | 1.5 (0.6) | 2.4 (0.6) | 2.0 (0.6) | 1.9 (0.6) | 0.618 |
| Constipation | 0.772 | 1.6 (0.6) | 1.9 (0.6) | 2.0 (0.6) | 2.6 (0.6) | 2.7 (0.6) | 3.0 (0.6) | 0.261 |
| # of bowel movements, count | 0.519 | 1.8 (0.2) | 1.6 (0.2) | 1.6 (0.2) | 1.3 (0.2) | 1.6 (0.2) | 1.6 (0.2) | 0.572 |
| Notes: SE: Standard error. #: number. Phase 1: Baseline week; Phase 2: The period of dosages ramping up (11 days); Phase 3: full-dose phase (10 days). IBS-SSS^d^: refers to all five rating questionnaires adopted from original ‘Irritable Bowel Syndrome -Severity Score System’, and can also generate a global score ranging from 0–500. GI symptoms were self-rated from 0 to 10, 10 indicated the worst severity of the symptoms. Linear Mixed Modelling was used to assess differences between- and within-groups with BMI = 22.8639 kg/m^2^, age = 37.20 y included as covariates in the model. No overall interaction significance was noted. | | | | | | | | |

In the ur gut® group, the mean score of gas increased from 3.7 at Phase 1 and 4.3 at Phase 2, and then declined to 3.9 at Phase 3, without statistical significance noted (Supplementary Table 2). This aligned with anecdotal self-report of participants taking ur gut® that they experienced bloating/more gas during the first few days (up to one week at most), which then was shown to revert to usual patterns. The score of gas was stable, 3.6, 3.4 and 3.7, respectively throughout the study in the placebo group (Supplementary Table 2).

Supplementary Table 3. Bowel habits and faeces samples by group and timepoint

| Faeces samples and bowel movements | Group × Time Interaction P | ur gut® Group, n=13  Mean (SE) | | | Placebo Group, n=13  Mean (SE) | | | Between-group P |
| --- | --- | --- | --- | --- | --- | --- | --- | --- |
|  |  | T1 | T2 | T2-T1 | T1 | T2 | T2-T1 |  |
| Stool weight, g | 0.461 | 155.0 (33.5) | 149.1 (33.5) | -5.8 (40.8) | 219.4 (33.5) | 170.3 (33.5) | -49.2 (40.8) | 0.266 |
| Bristol Stool Chart | 0.844 | 4.4 (0.4) | 4.2 (0.4) | -0.2 (0.5) | 4.4 (0.4) | 4.1 (0.4) | -0.3 (0.5) | 0.905 |
| # of bowel movements ^1^ | 0.281 | 1.8 (0.2) | 1.5 (0.2) | -0.3 (0.2) | 1.4 (0.2) | 1.4 (0.2) | 0 (0.2) | 0.418 |
| Note: T: Timepoint. SE: standard error. Linear Mixed Modelling was used to assess differences between- and within-groups with BMI= 22.90 and age = 37.15 included as covariates in the model. ^1^ The variable was the average of number of bowel movements during the last 3 days of each timepoint. No significance was noted. | | | | | | | | |

Supplementary Table 4. Daily intake of dietary micronutrients, FODMAP, and five food groups by group and timepoint

(Column title with *indicates values from both diet + supplement; values in bold indicate significance, ^ indicates FDR significance founded)

| Nutrients, unit | Group × Time Interaction | | | ur gut® Group, n=13  Mean (SE) | | | | | Placebo Group, n=13  Mean (SE) | | | | | Between-group | |
| --- | --- | --- | --- | --- | --- | --- | --- | --- | --- | --- | --- | --- | --- | --- | --- |
|  | P | | P* | T1 | T2 | T2-T1 | T2* | T2*-T1 | T1 | T2 | T2-T1 | T2* | T2*-T1 | P | P* |
| Diet Quality Index | 0.579 | | | 70.3 (3.9) | 70.8  (3.9) | 0.5  (4.1) |  |  | 75.5 (3.9) | 72.8 (3.9) | -2.7 (4.1) |  |  | 0.442 | |
| Macronutrients |  | |  |  |  |  |  |  |  |  |  |  |  |  |  |
| Food Weight, g | 0.287 | | 0.285 | 2547  (307) | 2141 (307) | **-407 (193)** | 2167 (307) | -380 (193) | 2526 (307) | 2416 (307) | -110 (193) | 2444 (307) | -82 (193) | 0.761 | 0.759 |
| Energy, kJ | **0.049** | | **0.032^** | 8271 (752) | 7045 (752) | -1226 (777) | 7205 (752) | -1066 (777) | 7662 (752) | 8712 (752) | 1051 (777) | 9091 (752) | 1430 (777) | 0.567 | 0.491 |
| Protein, g | **0.004** | | **0.004^** | 98.1 (10.3) | 79.9 (10.3) | **-18.2 (6.5)** | 80.4 (10.3) | **-17.7 (6.5)** | 80.3 (10.3) | 91.5 (10.3) | 11.3 (6.5) | 92.1 (10.3) | 11.8 (6.5) | 0.825 | 0.824 |
| Total fat, g | 0.105 | | 0.107 | 83.4 (9.9) | 70.8  (9.9) | -12.5 (11.6) | 71.1 (9.9) | -12.2 (11.6) | 79.7  (9.9) | 94.9 (9.9) | 15.2 (11.6) | 95.1 (9.9) | 15.4 (11.6) | 0.380 | 0.384 |
| Saturated fat, g | 0.125 | | 0.127 | 28.0 (4.8) | 25.0  (4.8) | -2.9 (6.2) | 25.1 (4.8) | -2.8  (6.2) | 25.8  (4.8) | 36.7 (4.8) | 10.9 (6.2) | 36.8 (4.8) | 10.9 (6.2) | 0.365 | 0.368 |
| Carbohydrate, g | 0.179 | | 0.172 | 161.9 (18.9) | 140.6 (18.9) | -21.3 (21.2) | 158.7 (18.9) | -3.2 (21.2) | 184.5 (18.9) | 204.7 (18.9) | 20.2 (21.2) | 223.6 (18.9) | 39.1 (21.2) | 0.063 | 0.061 |
| Sugars, g | 0.364 | | | 61.4 (13.9) | 55.0 (13.9) | -6.4 (15.9) |  |  | 66.7 (13.9) | 81.2 (13.9) | 14.4 (15.9) |  |  | 0.340 | |
| Starch, g | 0.225 | | 0.052 | 98.1 (11.7) | 84.1 (11.7) | -14 (11) | 90.2 (11.6) | -8.0 (10.9) | 117.4 (11.7) | 122.8 (11.7) | 5.5  (11.0) | 140.9 (11.6) | **23.5 (10.9)** | 0.059 | **0.024** |
| Dietary fibre, g | **0.016** | | 0.059 | 20.2 (2.5) | 16.4 (2.5) | **-3.8 (1.8)** | 33.1 (2.4) | **12.9**  **(1.8)** | 21.2  (2.5) | 24.1 (2.5) | 2.9 (1.8) | 29.0  (2.4) | **7.8 (1.8)** | 0.197 | 0.625 |
| Resistant Starch, g | 0.106 | **<0.001^** | | 2.0 (0.3) | 1.6 (0.3) | -0.3 (0.2) | 9.5 (0.3) | **7.6**  **(0.2)** | 2.3  (0.3) | 2.4 (0.3) | 2.3 (0.3) | 2.4 (0.3) | 0.1  (0.2) | 0.232 | **<0.001** |
| Alcohol, g | 0.714 | | | 19.8 (7.4) | 17.8 (7.4) | -2.0 (1.6) |  |  | 3.4  (7.4) | 2.3 (7.4) | -1.1 (1.6) |  |  | 0.139 | |
| A percentage energy from the macronutrients | | | | | |  |  |  |  |  |  |  |  |  |  |
| kJ from protein, % | 0.372 | | 0.486 | 20.4 (1.5) | 19.6  (1.5) | -0.7 (1.3) | 19.3 (1.5) | -1.1 (1.3) | 17.6  (1.5) | 18.6 (1.5) | 1.0 (1.3) | 17.9 (1.5) | 0.3  (1.3) | 0.341 | 0.282 |
| kJ from fat, % | 0.711 | | 0.956 | 38.2 (2.4) | 37.7  (2.4) | -0.5 (2.0) | 37.0 (2.4) | -1.2 (2.0) | 37.9  (2.4) | 38.4 (2.4) | 0.5 (2.0) | 36.8 (2.4) | -1.1 (2.0) | 0.951 | 0.936 |
| kJ from saturated fat, % | 0.331 | | 0.433 | 12.8 (1.1) | 13.3  (1.1) | 0.5 (1.1) | 13.1 (1.1) | 0.2  (1.1) | 12.0  (1.1) | 14.1 (1.1) | 2.1 (1.1) | 13.5 (1.1) | 1.5  (1.1) | 0.992 | 0.893 |
| Cont. |  | | |  | | | | |  | | | | |  | |
| Nutrients, unit | Group × Time Interaction | | | ur gut® Group, n=13  Mean (SE) | | | | | Placebo Group, n=13  Mean (SE) | | | | | Between-group | |
|  | P | | P* | T1 | T2 | T2-T1 | T2* | T2*-T1 | T1 | T2 | T2-T1 | T2* | T2*-T1 | P | P* |
| kJ from carbohydrate, % | 0.353 | | 0.629 | 31.6 (2.1) | 33.2  (2.1) | 1.6 (2.0) | 33.9 (2.1) | 2.3  (2.0) | 40.5  (2.1) | 39.4 (2.1) | -1.1 (2.0) | 41.4 (2.1) | 0.9  (2.0) | **0.008** | **0.004** |
| kJ from added sugar, % | 0.765 | | | 4.5 (1.4) | 4.1  (1.4) | -0.4 (1.3) |  |  | 7.1  (1.4) | 7.2 (1.4) | 0.1 (1.3) |  |  | 0.122 | |
| FODMAP | | | | | | | | | | | | | | | |
| Total FODMAP, g | 0.452 | | | 14.8 (3.3) | 8.8 (3.3) | -5.9 (3.1) |  |  | 13.0 (3.3) | 10.5 (3.3) | -2.5 (3.1) |  |  | 0.995 | |
| FODMAP without lactose, g | 0.579 | | | 7.1 (1.1) | 5.3 (1.1) | -1.8 (0.9) |  |  | 6.1 (1.1) | 5.0 (1.1) | -1.1 (0.9) |  |  | 0.670 | |
| Lactose, g | 0.571 | | | 7.7 (3.1) | 3.5 (3.1) | -4.1 (3.3) |  |  | 6.9 (3.1) | 5.5 (3.1) | -1.4 (3.3) |  |  | 0.874 | |
| Oligosaccharides, g | 0.584 | | | 3.6 (0.5) | 2.7 (0.5) | -1.0 (0.4) |  |  | 3.8 (0.5) | 3.2 (0.5) | -0.6 (0.4) |  |  | 0.642 | |
| Fructo-oligosaccharides, g | 0.866 | | | 2.5 (0.4) | 2.0 (0.4) | -0.5 (0.3) |  |  | 2.9 (0.4) | 2.4 (0.4) | -0.6 (0.3) |  |  | 0.446 | |
| Galacto-oligosaccharides, g | 0.244 | | | 1.1 (0.2) | 0.6 (0.2) | -0.4 (0.2) |  |  | 0.8 (0.2) | 0.8 (0.2) | -0.1 (0.2) |  |  | 0.886 | |
| Excess fructose, g | 0.076 | | | 0.0 (0.1) | 0.4 (0.1) | 0.4 (0.2) |  |  | 0.2 (0.1) | 0.0 (0.1) | -0.2 (0.2) |  |  | 0.477 | |
| Total polyols, g | 0.373 | | | 3.5 (0.8) | 2.3 (0.8) | -1.2 (0.7) |  |  | 2.2 (0.8) | 1.9 (0.8) | -0.3 (0.7) |  |  | 0.420 | |
| Food Groups | | | | | | | | | | | | | | | |
| Grains, serve | 0.052 | | **0.004** | 4.9 (0.7) | 3.9 (0.7) | -1.1 (0.7) | 3.9 (0.7) | -1.1  (0.7) | 5.4  (0.7) | 6.2 (0.7) | 0.9 (0.7) | 7.3 (0.7) | **1.9**  **(0.7)** | 0.101 | **0.027** |
| Refined, serve | 0.156 | | **0.018** | 3.9 (0.7) | 3.2 (0.7) | -0.8 (0.7) | 3.2 (0.7) | -0.8  (0.7) | 3.9  (0.7) | 4.6 (0.7) | 0.7 (0.7) | 5.7 (0.7) | **1.8**  **(0.7)** | 0.383 | 0.131 |
| Wholegrains, serve | 0.368 | | | 1.0 (0.4) | 0.7 (0.4) | -0.3 (0.3) |  |  | 1.5  (0.4) | 1.6 (0.4) | 0.1 (0.3) |  |  | 0.126 | |
| Fruit, serve | 0.991 | | | 0.9 (0.3) | 0.9 (0.3) | -0.1 (0.2) |  |  | 0.8 (0.3) | 0.8 (0.3) | -0.1 (0.2) |  |  | 0.763 | |
| Vegetables, serve | 0.846 | | | 4.9 (0.7) | 4.3 (0.7) | -0.7 (0.6) |  |  | 4.0 (0.7) | 3.5 (0.7) | -0.5 (0.6) |  |  | 0.396 | |
|  |  | | |  |  |  |  |  |  |  |  |  |  |  | |
| Cont. |  | | |  | | | | |  | | | | |  | |
| Nutrients, unit | Group × Time Interaction | | | ur gut® Group, n=13  Mean (SE) | | | | | Placebo Group, n=13  Mean (SE) | | | | | Between-group | |
|  | P | | P* | T1 | T2 | T2-T1 | T2* | T2*-T1 | T1 | T2 | T2-T1 | T2* | T2*-T1 | P | P* |
| Meat and meat alternatives, serve | **0.038** | | **0.036** | 3.2 (0.4) | 2.5 (0.4) | **-0.8**  **(0.3)** | 2.7 (0.4) | **-0.6**  **(0.3)** | 2.6 (0.4) | 2.6 (0.4) | 0.0 (0.3) | 2.8 (0.4) | 0.2  (0.3) | 0.575 | 0.585 |
| Dairy, serve | 0.629 | | | 1.4 (0.3) | 1.4 (0.3) | 0.1 (0.4) |  |  | 1.2 (0.3) | 1.5 (0.3) | 0.3 (0.4) |  |  | 0.906 | |
| Notes: FDR: False Discovery Rate. SE: Standard Error. T: Timepoint; T1, Timepoint 1 (baseline), T2, Timepoint 2. FODMAP: Fermentable Oligo-, Di-, Mono-saccharides And Polyols. Linear Mixed Modelling was used to assess differences between- and within-groups with 22.9076, and age = 37.15. included as covariates in the model.  Between-group difference (Group Placebo - Group ur gut®): RS -3.4 (0.4), P < 0.001. | | | | | | | | | | | | | | | |

For the result of Food Groups intake, grain and refined grain showed marginal interaction effects (P = 0.004 and 0.018, respectively, both FDR-adjusted P > 0.05) (Supplementary Table 4). The placebo group experienced significant increases in their grain intake, while it did not significantly change in the ur gut® group at T2 or T2*. In contrast, for the serve of protein foods, namely meat and meat alternatives (interaction P = 0.038, FDR adjusted P > 0.05), this was reduced in the ur gut® group by 0.8 ± 0.3 serve/d from T1 to 2.5 serves/d at T2*, whereas it was maintained in the placebo group, which is aligned with changes in protein intake in grams (Supplementary Table 4).

Supplementary Table 5. Sleep questionnaire results by group and timepoint

| Questionnaires | Group × Time  Interaction P | Group ur gut®, n=13  Mean (SE) | | | Group Placebo, n=13  Mean (SE) | | | Between-group P |
| --- | --- | --- | --- | --- | --- | --- | --- | --- |
|  |  | T1 | T2 | T2-T1 | T1 | T2 | T2-T1 |  |
| PSQI | 0.194 | 6.0 (1.0) | 6.2 (1.0) | 0.3 (0.6) | 7.7 (1.0) | 7.0 (1.0) | -0.8 (0.6) | 0.359 |
| ESS | 0.866 | 6.0 (1.2) | 5.9 (1.3) | -0.2 (0.9) | 6.1 (1.2) | 5.7 (1.2) | -0.4 (0.9) | 0.986 |
| ISI | 0.508 | 7.8 (1.7) | 8.0 (1.7) | 0.1 (1.2) | 10.2 (1.7) | 9.2 (1.7) | -1.0 (1.2) | 0.414 |
| SHI | 0.270 | 13.4 (1.9) | 13.1 (1.9) | -0.3 (1.0) | 18.1 (1.9) | 16.2 (1.9) | -1.8 (1.0) | 0.152 |
| RSQ-W | 0.340 | 51.2 (5.8) | 55.1 (6.0) | 3.9 (4.5) | 44.6 (5.8) | 42.5 (5.8) | -2.1 (4.3) | 0.225 |
| Notes: T: Timepoint; SE: Standard error; PSQI: The Pittsburgh Sleep Quality Index; ESS: The Epworth Sleepiness Scale; ISI: The Insomnia Severity Index; SHI: The Sleep Hygiene Index; RSQ-W: The Restorative Sleep Questionnaire weekly version; Linear Mixed Modelling was used to assess differences between- and within-groups with BMI = 22.9049, age = 37.27 included as covariates in the model. | | | | | | | | |

Supplementary Table 6. Results of mental health questionnaires by group and timepoint

| Questionnaires | Group × Time  Interaction P | Group ur gut®, n=13  Mean (SE) | | | Group Placebo, n=13  Mean (SE) | | | Between-group P |
| --- | --- | --- | --- | --- | --- | --- | --- | --- |
|  |  | T1 | T2 | T2-T1 | T1 | T2 | T2-T1 |  |
| DASS21-D^1^ | 0.084 | 5.0 (2.0) | 1.8 (2.1) | -3.2 (1.6) ^2^ | 8.6 (2.0) | 9.4 (2.0) | 0.8 (1.5) | 0.050 |
| DASS21-A | 0.394 | 6.4 (1.5) | 4.7 (1.5) | -1.7 (1.5) | 7.1 (1.5) | 7.2 (1.5) | 0.1 (1.4) | 0.419 |
| DASS21-S | 0.375 | 12.5 (2.3) | 10.3 (2.3) | -2.1 (1.3) | 15.3 (2.3) | 14.9 (2.3) | -0.5 (1.3) | 0.240 |
| VSI* | 0.034 | 46.3 (3.1) | 38.9 (3.1) | -7.4 (2.6) ^3^ | 43.1 (3.1) | 44 (3.1) | 0.8 (2.6) | 0.810 |
| Notes: T: Timepoint; SE: Standard error; DASS21: The 21-item Depression Anxiety Stress Scale; VSI: The Visceral Sensitivity Index; Linear Mixed Modelling was used to assess differences between- and within-groups with BMI = 22.9049, age = 37.27 included as covariates in the model.  ^1^ T2 Group mean difference (ur gut® - placebo): -7.5(2.9), P = 0.015.  ^2^ P = 0.055.  ^3^ P = 0.010.  * The covariate of age presented significance in the modeling of VSI: estimate (SE)= -0.6 (0.2), P = 0.005. | | | | | | | | |

Supplementary Table 7. Results of quality of life questionnaires by group and timepoint

| Questionnaires | Group × Time  Interaction P | Group ur gut®, n=13  Mean (SE) | | | Group Placebo, n=13  Mean (SE) | | | Between-group P |
| --- | --- | --- | --- | --- | --- | --- | --- | --- |
|  |  | T1 | T2 | T2-T1 | T1 | T2 | T2-T1 |  |
| WHO-5 ^1^ | 0.169 | 65.6 (5.1) | 73.0 (5.2) | 7.4 (3.6)^2^ | 54.5 (5.1) | 54.9 (5.1) | 0.3 (3.5) | 0.044 |
| IBS-QOL^3^ * | 0.259 | 44.4 (3.7) | 49.9 (3.8) | 5.5 (3.4) | 40.0 (3.7) | 40.0 (3.7) | 0 (3.3) | 0.147 |
| Dysphoria* | 0.963 | 51.3 (4.8) | 54.6 (4.9) | 3.3 (3.7) | 40.9 (4.8) | 43.9 (4.8) | 3.1 (3.5) | 0.108 |
| Interference with activity* | 0.261 | 42.4 (4.7) | 47.5 (4.8) | 5.1 (4.3) | 44.1 (4.7) | 42.3 (4.7) | -1.8 (4.1) | 0.772 |
| Body image ^4^ | 0.214 | 41.9 (5.4) | 47.3 (5.6) | 5.4 (5.0) | 32.9 (5.4) | 29.4 (5.4) | -3.5 (4.9) | 0.064 |
| Health worry | 0.146 | 46.5 (5.4) | 53.8 (5.5) | 7.4 (5.5) | 47.0 (5.4) | 42.9 (5.4) | -4.1 (5.3) | 0.444 |
| Food avoidance* | 0.672 | 22.4 (4.5) | 31.0 (4.7) | 8.5 (5.7) | 19.2 (4.5) | 24.4 (4.5) | 5.1 (5.5) | 0.347 |
| Social reaction | 0.397 | 48.7 (5.6) | 52.4 (5.7) | 3.7 (4.7) | 41.9 (5.6) | 39.9 (5.6) | -1.9 (4.6) | 0.195 |
| Sexual ^5^ | 0.019 | 42.3 (6.2) | 54.8 (6.3) | 12.5 (4.9)^6^ | 43.9 (6.2) | 39.3 (6.2) | -4.6 (4.7) | 0.400 |
| Relationship* | 0.942 | 49.0 (5.5) | 54.7 (5.7) | 5.7  (5.8) | 46.1 (5.5) | 51.2 (5.5) | 5.1 (5.6) | 0.642 |
| Notes: T: Timepoint; SE: Standard error; WHO-5: WHO (Five) Well-Being Index; IBS-QOL: Irritable Bowel Syndrome Quality of Life; Linear Mixed Modelling was used to assess differences between- and within-groups with BMI = 22.9049, age = 37.27 included as covariates in the model. No False Discovery Rate adjusted P was significant.  ^1^ T2 group mean difference (ur gut® - placebo): 18.1(7.3), P=0.019.  ^2^ P = 0.051.  ^3^ T2 group mean difference (ur gut® - placebo): 9.9 (5.4) P =0.073.  ^4^ T2 group mean difference (ur gut® - placebo): 17.9 (7.8), P = 0.028.  ^5^ T2 group mean difference (ur gut® - placebo): 15.5 (8.9), P = 0.09.  ^6^ P = 0.018.  * The covariate of age presented significance in the modeling of  IBS-QoL*: estimate (SE)=1.0 (0.3), P = 0.002;  Dysphoria*: estimate (SE)=0.6 (0.3), P = 0.037;  Interference with activity*: estimate (SE)=0.7(0.2), P = 0.004;  Food avoidance*: estimate (SE)=0.6 (0.2), P = 0.023;  Relationship*: estimate (SE)=0.9 (0.3), P = 0.010. | | | | | | | | |

Supplementary Table 8. SCFA concentration and excretion amount comparison by group and timepoint

| Faecal SCFA | Group × Time  Interaction P | Group ur gut®, n=13  Mean (SE) | | | | Group Placebo, n=13  Mean (SE) | | | | Between-group P |
| --- | --- | --- | --- | --- | --- | --- | --- | --- | --- | --- |
|  |  | T1 | T2 | T2-T1 | Change (%) | T1 | T2 | T2-T1 | Change (%) |  |
| Stool weight, g | 0.461 | 155.0 (33.5) | 149.1 (33.5) | -5.8  (40.8) | -3.7 | 219.4 (33.5) | 170.3 (33.5) | -49.2 (40.8) | -22.4 | 0.266 |
| Concentration, μmol/g | | |  |  |  |  |  |  |  |  |
| Total SCFA | 0.569 | 96.7 (8.4) | 97.4 (8.4) | 0.7 (8.8) | 0.7 | 85.9 (8.4) | 93.7 (8.4) | 7.9 (8.8) | 9.2 | 0.483 |
| Acetate | 0.442 | 51.5 (4.8) | 55.2 (4.8) | 3.7 (5.1) | 7.2 | 48.1 (4.8) | 57.4 (4.8) | 9.3 (5.1) | 19.3 | 0.917 |
| Propionate | 0.147 | 20.3 (1.7) | 16.5 (1.7) | -3.9 (2.3) | -19.2 | 15.6 (1.7) | 16.6 (1.7) | 1.0 (2.3) | 6.4 | 0.201 |
| Butyrate | 0.400 | 16.9 (2.3) | 18.6 (2.3) | 1.7 (2.1) | 10.1 | 15 (2.3) | 14.1 (2.3) | -0.9 (2.1) | -6.0 | 0.288 |
| Isovalerate | 0.476 | 3.5 (0.6) | 3.1 (0.6) | -0.3 (0.6) | -8.6 | 3.3 (0.6) | 2.4 (0.6) | -0.9 (0.6) | -27.3 | 0.542 |
| Isobutyrate | 0.763 | 2.2 (0.3) | 1.7 (0.3) | -0.5 (0.3) | -22.7 | 2.0 (0.3) | 1.7 (0.3) | -0.3 (0.3) | -15 | 0.789 |
| Valerate | 0.567 | 2.4 (0.3) | 2.3 (0.3) | -0.1 (0.3) | -4.2 | 1.9 (0.3) | 1.6 (0.3) | -0.3 (0.3) | -15.8 | 0.076 |
| Excretion amount, mmol | | |  |  |  |  |  |  |  |  |
| Total SCFA | 0.820 | 15.4 (3.2) | 14.2 (3.2) | -1.2 (4.4) | -7.7 | 18.6 (3.2) | 16 (3.2) | -2.6 (4.4) | -14.1 | 0.468 |
| Acetate | 0.876 | 8.3 (2.0) | 8.1 (2.0) | -0.2 (2.4) | -2.1 | 10.7 (2.0) | 10.0 (2.0) | -0.7 (2.4) | -6.7 | 0.345 |
| Propionate | 0.765 | 3.3 (0.7) | 2.4 (0.7) | -1.0 (1.0) | -28.6 | 3.5 (0.7) | 3.0 (0.7) | -0.5 (1.0) | -14.4 | 0.576 |
| Butyrate ^1^ | 0.418 | 2.7 (0.6) | 2.8 (0.6) | 0.1 (0.8) | 2.1 | 3.0 (0.6) | 2.2 (0.6) | -0.9 (0.8) | -28.5 | 0.790 |
| Isovalerate | 0.278 | 0.5 (0.1) | 0.4 (0.1) | 0.0 (0.1) | -9.1 | 0.6 (0.1) | 0.3 (0.1) | -0.3 (0.1) | -44.1 | 0.912 |
| Isobutyrate | 0.647 | 0.3 (0.1) | 0.3 (0.1) | 0.0 (0.1) | -16.1 | 0.4 (0.1) | 0.3 (0.1) | -0.1 (0.1) | -27.8 | 0.427 |
| Valerate ^2^ | 0.381 | 0.4 (0.1) | 0.3 (0.1) | 0.0 (0.1) | -7.7 | 0.4 (0.1) | 0.2 (0.1) | -0.2 (0.1) | -41.4 | 0.573 |
| Notes:  T: Timepoint; SE: Standard error; SCFA: Short Chain Fatty Acid. Linear Mixed Modelling was used to assess differences between- and within-groups with BMI = 22.9077, age = 37.15 included as covariates in the model.  ^1^ The fixed effects of the mixed model analysis of butyrate shows a significant effect of BMI with estimate (SE) = 0.2 (0.1), P=0.044.  ^2^ The fixed effects of the mixed model analysis of valeric acid shows a significant effect of BMI with estimate (SE) = 0.02 (0.01), P=0.021. | | | | | | | | | | |

Supplementary Table 9. SCFA concentration and excretion amount, and faecal weight in the two subgroups of the ur gut® group by timepoint

| Stool weight, number of bowel movements and SCFA | Group × Time  Interaction P | Group ur gut® _LOW_, n = 5  Mean (SE) | | | | Group ur gut® _HIGH_, n = 8  Mean (SE) | | | | Between-group P |
| --- | --- | --- | --- | --- | --- | --- | --- | --- | --- | --- |
|  |  | T1 | T2 | T2-T1 | Change (%) | T1 | T2 | T2-T1 | Change (%) |  |
| Stool weight, g/d | **0.042¹** | 77.5 (32.9) | 161.9 (32.9) | 84.4 (50.1) | 108.9% | 205.6 (25.7) | 143.3 (25.7) | -62.2 (39.6) | -30.3% | 0.088 |
| # of bowel movements, count | 0.222 | 1.3 (0.3) | 1.3 (0.3) | 0  (0.4) | 0% | 2.1 (0.3) | 1.4 (0.3) | -0.6 (0.3) | -42.90% | 0.233 |
| Concentration, μmol/g | |  |  |  |  |  |  |  |  |  |
| Total SCFA | 0.164 | 84.6 (13.3) | 103.3 (13.3) | 18.7  (15.4) | 22.1% | 104.5 (10.3) | 93.9 (10.3) | -10.6  (12.2) | 118.4% | 0.727 |
| Acetate | 0.121 | 43.0 (8.0) | 58.2 (8.0) | 15.1 (8.7) | 35.1% | 57.0 (6.2) | 53.5 (6.2) | -3.4 (6.9) | -6.0% | 0.614 |
| Propionate | 0.532 | 18.4 (3.0) | 16.9 (3.0) | -1.5 (4.6) | -8.2% | 21.5 (2.3) | 16.2 (2.3) | -5.3 (3.6) | -24.7% | 0.661 |
| Butyrate | **0.029** | 13.3 (3.0) | 20.1 (3.0) | 6.8 (2.6) | **51.1%^2^** | 19.1 (2.3) | 17.6 (2.3) | -1.5 (2.1) | -7.9% | 0.643 |
| Isovalerate | 0.316 | 4.5 (1.0) | 3.3 (1.0) | -1.2 (1.1) | -26.7% | 2.8 (0.8) | 3.0 (0.8) | 0.2 (0.9) | 7.1% | 0.652 |
| Isobutyrate | 0.337 | 2.6 (0.5) | 1.6 (0.5) | -0.9 (0.6) | -34.6% | 1.9 (0.4) | 1.7 (0.4) | -0.2 (0.5) | -10.5% | 0.397 |
| Valerate | 0.249 | 2.7 (0.5) | 3.2 (0.5) | 0.5 (0.5) | 18.5% | 2.2 (0.4) | 1.8 (0.4) | -0.4 (0.4) | -18.2% | 0.114 |
| Excretion amount, mmol | |  |  |  |  |  |  |  |  |  |
| Total SCFA | **0.015** | 5.9 (3.8) | 17.1 (3.8) | 11.2 (5.5) | 189.8% | 21.6 (3) | 12.6 (3) | -9.0 (4.3) | -41.7% | 0.154 |
| Acetate | **0.017** | 3.1 (2.2) | 11.6 (1.7) | 8.6 (2.8) | **277.4%^3^** | 9.7 (2.2) | 7.2 (1.7) | -2.4 (2.8) | -24.7% | 0.169 |
| Propionate | **0.050** | 1.2 (0.9) | 2.7 (0.9) | 1.6 (1.4) | 133.3% | 4.7 (0.7) | 2.2 (0.7) | -2.5 (1.1) | -53.2% | 0.063 |
| Butyrate | **0.006** | 0.9 (0.8) | 3.5 (0.8) | 2.6 (0.9) | **288.9%^2^** | 3.9 (0.6) | 2.4 (0.6) | -1.5 (0.7) | -38.5% | 0.616 |
| Isovalerate | 0.213 | 0.4 (0.2) | 0.5 (0.2) | 0.1 (0.2) | 25.0% | 0.5 (0.1) | 0.4 (0.1) | -0.2 (0.1) | -40.0% | 0.346 |
| Isobutyrate | 0.124 | 0.2 (0.1) | 0.3 (0.1) | 0.1 (0.1) | 50.0% | 0.4 (0.1) | 0.2 (0.1) | -0.1 (0.1) | -25.0% | 0.959 |
| Valerate | 0.032 | 0.2 (0.1) | 0.5 (0.1) | 0.3 (0.2) | 150.0% | 0.5 (0.1) | 0.2 (0.1) | -0.2 (0.1) | -40.0% | 0.905 |
| Note:  ur gut® _LOW_: participants in the ur gut® group with a T1 faecal output lower than 150 g/d.  ur gut® _HIGH_: participants in the ur gut® group with a T1 faecal output higher than 150 g/d.  Linear Mixed Modelling was used to assess differences between- and within-groups with BMI = 22.9731, age = 37.62 included as covariates in the model.  All False Discovery Rate > 0.05.  ¹ at T1, the between-group difference, 128.1 (42.9) was significant, P = 0.007.  ^2^ P < 0.05. ^3^ P < 0.01. | | | | | | | | | | |

Supplementary Table 10. SCFA concentration and excretion amount, and faecal weight in the two subgroups of the placebo group by timepoint

| Stool weight, number of bowel movements and SCFA | Group × Time  Interaction P | Group Placebo _LOW_, n=6  Mean (SE) | | | | Group Placebo _HIGH_, n=7  Mean (SE) | | | | Between-group P |
| --- | --- | --- | --- | --- | --- | --- | --- | --- | --- | --- |
|  |  | T1 | T2 | T2-T1 | Change (%) | T1 | T2 | T2-T1 | Change (%) |  |
| Stool weight, g/d | **0.036** | 119.9 (63.1) | 169.1 (63.1) | 49.2 (56.2) | 41.0% | 302.2 (57.4) | 168.8 (57.4) | -133.4 (52.1)¹ | -44.1% | 0.318 |
| # of bowel movements, count | 0.103 | 1.0 (0.4) | 1.5 (0.4) | 0.5 (0.3) | 50.0% | 2.0 (0.4) | 1.7 (0.4) | -0.3 (0.3) | -15.0% | 0.395 |
| Concentration, μmol/g | |  |  |  |  |  |  |  |  |  |
| Total SCFA | 0.749 | 70.3 (15.2) | 75.4 (15.2) | 5.1 (11.4) | 7.3% | 99.0 (13.8) | 109.2 (13.8) | 10.2 (10.6) | 10.3% | 0.181 |
| Acetate | 0.432 | 38.5 (7.8) | 44.0  (7.8) | 5.5 (6.5) | 14.3% | 56.1 (7.1) | 68.8 (7.1) | 12.6 (6.0) | 22.5% | 0.081 |
| Propionate | 0.254 | 12.9 (2.9) | 15.9 (2.9) | 3.1 (2.4) | 24.0% | 18.0 (2.6) | 17.2 (2.6) | -0.8 (2.2) | -4.4% | 0.443 |
| Butyrate | 0.716 | 12.0 (4.9) | 10.1 (4.9) | -1.8 (3.5) | -15.0% | 17.6 (4.4) | 17.5 (4.4) | -0.1 (3.2) | -0.6% | 0.915 |
| Isovalerate | 0.999 | 3.5 (1.2) | 2.5 (1.2) | -0.9 (0.6) | -25.7% | 3.1 (1.1) | 2.2 (1.1) | -0.9 (0.6) | -29.0% | 0.373 |
| Isobutyrate | 0.729 | 2.0 (0.6) | 1.5 (0.6) | -0.5 (0.4) | -25.0% | 2.0 (0.5) | 1.7 (0.5) | -0.2 (0.4) | -10.0% | 0.834 |
| Valerate | 0.802 | 1.5 (0.4) | 1.3 (0.4) | -0.2 (0.5) | -13.3% | 2.2 (0.4) | 1.9 (0.4) | -0.4 (0.4) | -18.2% | 0.219 |
| Excretion amount, mmol | |  |  |  |  |  |  |  |  |  |
| Total SCFA | 0.082 | 7.3 (5.2) | 12.9 (5.2) | 5.6 (5.9) | 76.7% | 28.0 (4.8) | 18.4 (4.8) | -9.7 (5.4) | -34.6% | 0.077 |
| Acetate | 0.107 | 4.0 (3.6) | 7.5 (3.6) | 3.5 (3.3) | 87.5% | 16.3 (3.3) | 11.9 (3.3) | -4.3 (3.0) | -26.4% | 0.119 |
| Propionate | 0.122 | 1.6 (1.2) | 2.8 (1.2) | 1.2 (1.4) | 75.0% | 5.2 (1.1) | 3.2 (1.1) | -2.0 (1.3) | -38.5% | 0.239 |
| Butyrate | 0.063 | 1.1 (0.8) | 1.8 (0.8) | 0.7 (1.0) | 63.6% | 4.6 (0.7) | 2.4 (0.7) | -2.2 (0.9) | -47.8% | 0.104 |
| Isovalerate | 0.117 | 0.3 (0.2) | 0.3 (0.2) | 0.0 (0.2) | 0% | 0.9 (0.1) | 0.4 (0.1) | -0.5 (0.2) | -55.6% | 0.045 |
| Isobutyrate | **0.032** | 0.2 (0.1) | 0.3 (0.1) | 0.1 (0.1) | 50.0% | 0.5 (0.1) | 0.3 (0.1) | -0.3 (0.1) ¹ | -60.0% | 0.086 |
| Valerate | **0.041** | 0.2 (0.1) | 0.2 (0.1) | 0.1 (0.1) | 50.0% | 0.6 (0.1) | 0.2 (0.1) | -0.4 (0.1) ¹ | -66.7% | 0.069 |
| Note:  Placebo _LOW_: participants in the placebo group with a T1 faecal output lower than 150g/d.  Placebo _HIGH:_ participants in the placebo group with a T1 faecal output higher than 150g/d.  Linear Mixed Modelling was used to assess differences between- and within-groups with BMI = 22.8423, age = 36.69 included as covariates in the model.  ¹ P < 0.05  All False Discovery Rate P > 0.05. | | | | | | | | | | |

Supplementary Table 11. Serum biomarkers by group and timepoint

| Serum Biomarkers, unit | Group × Time  Interaction P | Group ur gut®, n=13  Mean (SE) | | | Group Placebo, n=13  Mean (SE) | | | Between-  group P |
| --- | --- | --- | --- | --- | --- | --- | --- | --- |
|  |  | T1 | T2 | T2-T1 | T1 | T2 | T2-T1 |  |
| IL-6 ^1^, pg/mL | 0.157 | 2.1 (0.7) | 3.1 (0.7) | 1.0 (0.4) | 1.0 (0.7) | 1.1 (0.7) | 0.1 (0.4) | 0.089 |
| hs-CRP ^1^, mg/L | 0.682 | 0.5 (0.3) | 0.6 (0.3) | 0.1 (0.4) | 0.9 (0.3) | 1.2 (0.3) | 0.3 (0.4) | 0.305 |
| Note: ^1^ Results were generated from 51 (98.1%) samples that were above the detection limit of the assay in IL-6; 46 (88.5%) in hs-CRP. Modelling was adjusted with BMI = 22.9608, and age = 37.27. | | | | | | | | |

Supplementary Table 12. The relative abundance and fold change of 26 genera by group and timepoint, and number of participants with the direction of change observed in the group at T2.

| Genus | ur gut® Group, n=13 | | | | Placebo Group, n=13 | | | |
| --- | --- | --- | --- | --- | --- | --- | --- | --- |
|  | T1 RA, % | T2 RA, % | fc  (T2/T1) | # of observations/  # of participants with the taxa | T1 RA, % | T2 RA, % | fc  (T2/T1) | # of observations/  # of participants with the taxa |
| Unclassified to genus | 22.9% | 17.4% | -1.3 | 10 / 13 | 25.9% | 23.2% | -1.1 | 7 / 13 |
| Blautia A | 20.1% | 12.8% | -1.6 | 10 / 13 | 18.2% | 16.4% | -1.1 | 7 / 13 |
| Bacteroides | 13.7% | 8.1% | -1.7 | 9 / 13 | 6.6% | 16.3% | 2.5 | 10 / 13 |
| Faecalibacterium | 8.6% | 8.8% | 1.0 | 5 / 13 | 8.9% | 10.3% | 1.1 | 7 / 13 |
| Ruminococcus E ³ | 2.1% | 11.3% | **5.5** | 12 / 13 | 1.6% | 0.6% | -2.7 | 9 / 13 |
| Anaerobutyricum | 2.5% | 1.5% | **-1.6** | 10 / 13 | 5.1% | 3.3% | -1.5 | 9 / 13 |
| Fusicatenibacter | 3.5% | 1.9% | -1.8 | 12 / 13 | 3.2% | 2.5% | -1.3 | 7 / 13 |
| Prevotella ¹ ^4^ | 1.3% | 7.9% | 5.9 | 7 / 13 | 0.03% | 0.03% | 1.0 | 7 / 13 |
| Bifidobacterium | 0.7% | 0.4% | -1.8 | 8 / 13 | 4.1% | 3.9% | -1.1 | 6 / 13 |
| Akkermansia | 1.4% | 3.7% | 2.6 | 7 / 13 | 1.5% | 0.8% | -1.8 | 9 / 13 |
| Methanobrevibacter A | 1.0% | 1.0% | -1.1 | 3 /12 | 2.3% | 3.0% | 1.3 | 5 / 12 |
| Roseburia | 1.1% | 2.0% | 1.9 | 7 /13 | 1.8% | 0.6% | -2.8 | 9 / 13 |
| Fusobacterium A ² | 0.000% | 0.002% | 4.9 | 3 /3 | 2.0% | 3.1% | 1.5 | 4 / 9 |
| Alistipes | 1.7% | 1.4% | -1.2 | 6 /13 | 0.7% | 0.9% | 1.2 | 5 / 13 |
| Phocaeicola ¹ ^4^ | 1.0% | 2.0% | 1.9 | 4 /13 | 0.6% | 0.6% | 1.1 | 7 / 12 |
| Clostridium | 1.0% | 2.6% | 2.7 | 8 /13 | 0.2% | 0.5% | 2.3 | 10 / 13 |
| Erysipelatoclostridium | 0.9% | 0.4% | -2.3 | 9 /13 | 1.2% | 1.0% | -1.2 | 9 / 13 |
| Lachnospira | 1.3% | 0.6% | -2.1 | 10 /13 | 0.7% | 0.3% | -2.2 | 7 / 13 |
| Coprococcus | 1.2% | 0.9% | -1.3 | 11 /13 | 0.3% | 0.3% | 1.2 | 4 / 13 |
| CAG-495 | 0.1% | 2.3% | 17.8 | 10 /11 | 0.0% | 0.1% | 2.7 | 7 / 9 |
| Dorea | 0.5% | 0.3% | -1.8 | 11 /13 | 1.1% | 0.4% | -2.6 | 8 / 12 |
| Acutalibacter | 0.3% | 0.2% | -1.5 | 8 /12 | 1.2% | 0.6% | -2.1 | 8 / 13 |
| Ruminococcus B | 0.1% | 0.04% | -2.4 | 7 /13 | 1.6% | 0.3% | -4.8 | 4 / 13 |
| Holdemanella ² | 1.1% | 0.4% | -2.6 | 10 /13 | 0.01% | 0.01% | 1.2 | 7 / 13 |
| CAG-279 | 0.3% | 1.1% | 4.3 | 7 /11 | 0.004% | 0.004% | 1.1 | 6 / 10 |
| UBA7173 | 0.02% | 0.01% | -1.6 | 3 /7 | 0.18% | 1.12% | 6.3 | 3 / 6 |
| Note: Genera listed are relatively abundant above 1% in either group or timepoint. RA: relative abundance; fc: Fold Change. T1: Timepoint 1 (baseline); T2: Timepoint 2 (Post-intervention). # of responders \ # Total observed: The number of responders to the change (positive or negative, indicated in the  ¹ T1 Between-group difference was significant, P <0.01.  ² T1 Between-group difference was significant, P <0.05.  ³ T2 Between-group difference was significant, P <0.01, FDR.P=0.0037.  ^4^ T2 Between-group difference was significant, P <0.05.  Text in bold indicates the difference within ur gut® group was significant P < 0.01.  All FDR.P values except one mentioned above were P > 0.05. | | | | | | | | |

Supplementary Table 13. Relative abundance of genera with significant difference between groups and within the group

| Genus | T1-Baseline | | | T2-End of Intervention | | |
| --- | --- | --- | --- | --- | --- | --- |
|  | ur gut® Group, n=13 | Placebo group, n=13 | P < 0.05 | ur gut® Group, n=13 | Placebo group, n=13 | P < 0.05 |
| Ruminococcus_E** | **2.0504%** | **1.6338%** |  | **11.3305%** | 0.6042% | **0.000** |
| Anaerobutyricum* | **2.5116%** | **5.0632%** |  | **1.5354%** | **3.3277%** |  |
| Prevotella | **1.3356%** | 0.0303% | 0.002 | **7.9221%** | 0.0317% | 0.026 |
| Fusobacterium_A | 0.0005% | **2.0247%** | 0.014 | 0.0023% | **3.0781%** |  |
| Phocaeicola | **1.0384%** | 0.5839% | 0.006 | **1.9801%** | 0.6322% | 0.019 |
| Holdemanella | **1.0905%** | 0.0086% | 0.016 | 0.4196% | 0.0105% |  |
| CAG-45 | 0.5972% | 0.0756% |  | 0.3470% | 0.2820% | 0.045 |
| Senegalimassilia | 0.0496% | 0.0230% | 0.001 | 0.0490% | 0.0310% |  |
| Lactonifactor | 0.0207% | 0.0300% | 0.048 | 0.0376% | 0.0070% |  |
| Firm-11 | 0.0265% | 0.0066% | 0.043 | 0.0368% | 0.0119% | 0.023 |
| CAG-873 | 0.0239% | 0.0001% |  | 0.0468% | 0 | 0.037 |
| Slackia_A | 0.0414% | 0 | 0.008 | 0.0105% | 0 | 0.037 |
| GCA-900066495 | 0.0007% | 0 |  | 0.0233% | 0.0123% | 0.049 |
| Desulfovibrio | 0.0002% | 0.0090% |  | 0 | 0.0260% | 0.037 |
| Catenibacterium* | 0.0152% | 0.0052% |  | 0.0016% | 0.0060% |  |
| QAMH01 | 0.0141% | 0 | 0.008 | 0.0005% | 0.0037% |  |
| CAG-313 | 0.0029% | 0.0018% |  | 0.0135% | 0 | 0.037 |
| Note: Genera listed in the table are those having significant differences between groups at T1 and T2, as well as within ur gut® group. No significance was observed within Placebo group. The relative abundance over 1% in the group was in bold. P value in bold indicates its False Discovery Rate (FDR) P values less than 0.01 (=0.00370). No rest of FDR.P value less than 0.05. Genera **: Within ur gut® group P<0.01; Genera * Within ur gut® group P < 0.05. | | | | | | |

Supplementary Table 14. The relative abundance and fold change of 42 ASV by group and timepoint, and number of participants with the direction of change observed in the group at T2.

| Amplicon sequence variant (ASV) | ur gut® Group, n=13 | | | | Placebo Group, n=13 | | | |
| --- | --- | --- | --- | --- | --- | --- | --- | --- |
|  | T1 RA, % | T2 RA, % | fc (T2/T1) | # of observations/  # of participants with the taxa | T1 RA,% | T2 RA,% | fc (T2/T1) | # of observations/  # of participants with the taxa |
| ASV 1* Blautia wexlerae | 10.1% | 5.0% | -2 | 12 / 13 | 11.0% | 9.8% | -1.1 | 6 / 13 |
| ASV 6* Bacteroides vulgatus | 4.1% | 1.6% | -2.5 | 7 / 13 | 2.2% | 4.7% | 2.1 | 7 / 12 |
| ASV 24* Bacteroides uniformis | 3.8% | 0.8% | -4.7 | 10 / 13 | 0.8% | 0.8% | -1.1 | 8 / 11 |
| ASV 2 Faecalibacterium prausnitzii C | 3.5% | 4.2% | 1.2 | 6 / 13 | 4.2% | 5.3% | 1.3 | 7 / 13 |
| ASV 8 Fusicatenibacter saccharivorans | 3.5% | 1.9% | -1.8 | 12 / 13 | 3.1% | 2.5% | -1.3 | 7 / 13 |
| ASV 3* Blautia massiliensis | 3.4% | 1.7% | -2 | 10 / 13 | 2.1% | 2.2% | 1.1 | 5 / 13 |
| ASV 5* Blautia faecis | 3.3% | 3.3% | 1 | 6 / 13 | 2.3% | 2.1% | -1.1 | 5 / 13 |
| ASV 7 Faecalibacterium prausnitzii J | 2.7% | 2.1% | -1.3 | 8 / 13 | 2.8% | 3.2% | 1.1 | 5 / 13 |
| ASV 4* Eubacterium rectale | 1.8% | 1.5% | -1.2 | 7 / 13 | 3.3% | 3.7% | 1.1 | 4 / 13 |
| ASV 14 Ruminococcus E ¹ ³ | 1.6% | 6.7% | **4.3** | 11 / 13 | 1.3% | 0.4% | -3.1 | 8 / 13 |
| ASV 20* Dorea longicatena | 1.5% | 0.9% | -1.7 | 10 / 13 | 1.2% | 0.8% | -1.6 | 10 / 13 |
| ASV 9* Phocaeicola dorei | 1.3% | 1.0% | -1.3 | 9 / 12 | 4.4% | 3.0% | -1.5 | 7 / 12 |
| ASV 35 Akkermansia | 1.3% | 0.2% | -5.3 | 5 / 9 | 0.1% | 0.3% | 4.5 | 4 / 12 |
| ASV 31* Bacteroides cellulosilyticus | 1.2% | 1.8% | 1.5 | 7 / 13 | 0.2% | 1.5% | 6.6 | 6 / 12 |
| ASV 49* Ruminococcus bicirculans | 1.2% | 0.6% | -2 | 7 / 13 | 0.2% | 0.2% | -1.3 | 8 / 13 |
| ASV 45* Roseburia inulinivorans | 1.1% | 0.3% | -3.6 | 7 / 11 | 0.2% | 0.3% | 1.4 | 4 / 13 |
| ASV 54* Bacteroides thetaiotaomicron ² | 1.1% | 0.4% | -2.7 | 8 / 13 | 0.2% | 0.4% | 1.8 | 7 / 13 |
| ASV 37 Phocaeicola¹ ³ | 1.0% | 2.0% | 1.9 | 4 / 13 | 0.6% | 0.2% | -3.3 | 6 / 11 |
| ASV 19 Methanobrevibacter A smithii A | 1.0% | 1.0% | -1.1 | 3 / 12 | 2.2% | 2.9% | 1.3 | 5 / 12 |
| ASV 22 Faecalibacterium | 1.0% | 1.4% | 1.4 | 8 / 13 | 0.9% | 0.7% | -1.4 | 7 / 13 |
| ASV 23 Anaerobutyricum hallii | 1.0% | 0.7% | -1.4 | 8 / 13 | 2.0% | 0.9% | -2.2 | 9 / 13 |
| ASV 26 Erysipelatoclostridium | 0.9% | 0.4% | -2.3 | 10 / 13 | 1.1% | 0.9% | -1.2 | 8 / 13 |
| ASV 30 Anaerobutyricum hallii A soehngenii | 0.8% | 0.4% | -1.9 | 10 / 13 | 1.2% | 1.1% | -1.1 | 8 / 13 |
| ASV 61 Clostridium sp001916075 | 0.7% | 1.8% | 2.7 | 8 / 13 | 0.1% | 0.2% | 3.6 | 8 / 12 |
| ASV 32 Roseburia | 0.6% | 1.3% | 2.3 | 7 / 12 | 1.5% | 0.3% | -4.5 | 7 / 13 |
| ASV 25 Bacteroides uniformis | 0.5% | 0.2% | -2.9 | 6 / 9 | 0.5% | 1.7% | 3.6 | 6 / 13 |
| Cont. |  |  |  |  |  |  |  |  |
| Amplicon sequence variant (ASV) | ur gut® Group, n=13 | | | | Placebo Group, n=13 | | | |
|  | T1 RA, % | T2 RA, % | fc (T2/T1) | # of observations/  # of participants with the taxa | T1 RA,% | T2 RA,% | fc (T2/T1) | # of observations/  # of participants with the taxa |
| ASV 25 Bacteroides uniformis | 0.5% | 0.2% | -2.9 | 6 / 9 | 0.5% | 1.7% | 3.6 | 6 / 13 |
| ASV 21 Ruminococcus E bromii A sp003526955 | 0.5% | 4.4% | 9.7 | 5 / 11 | 0.3% | 0.1% | -2.3 | 5 / 13 |
| ASV 47 Anaerobutyricum | 0.4% | 0.3% | -1.7 | 10 / 11 | 1.1% | 0.7% | -1.6 | 9 / 13 |
| ASV 11 Prevotella copri A ¹ | 0.4% | 3.6% | 8.8 | 6 / 13 | 0.02% | 0.01% | -1.2 | 6 / 13 |
| ASV 40* Bacteroides stercoris | 0.4% | 0.4% | 1.1 | 5 / 13 | 0.8% | 1.5% | 2 | 7 / 13 |
| ASV 16 Bifidobacterium sp900752285 | 0.3% | 0.2% | -1.7 | 7 / 12 | 2.7% | 3.0% | 1.1 | 5 / 10 |
| ASV 46 Acutalibacter sp003531055 | 0.3% | 0.2% | -1.5 | 8 / 12 | 1.1% | 0.5% | -2 | 8 / 13 |
| ASV 18 Prevotella copri A sp900546535 ² | 0.3% | 2.6% | 9.1 | 6 / 12 | 0.01% | 0.01% | 1.3 | 4 / 10 |
| ASV 41 Bacteroides | 0.2% | 0.3% | 1.3 | 6 / 11 | 0.5% | 1.0% | 1.9 | 5 / 11 |
| ASV 48 CAG-495 | 0.1% | 2.2% | 18.5 | 5 / 6 | 0.003% | 0.01% | 1.5 | 4 / 6 |
| ASV 12 Akkermansia muciniphila | 0.1% | 3.5% | 32.9 | 8 / 13 | 0.1% | 0.5% | 4.9 | 4 / 12 |
| ASV 28 Bacteroides | 0.1% | 0.2% | 1.7 | 4 / 12 | 0.3% | 2.2% | 8.3 | 6 / 12 |
| ASV 33 Bifidobacterium infantis | 0.08% | 0.09% | 1.2 | 5 / 13 | 1.3% | 0.6% | -2.1 | 6 / 12 |
| ASV 64 Ruminococcus B gnavus | 0.06% | 0.03% | -2.1 | 7 / 13 | 1.3% | 0.2% | -5.6 | 4 / 12 |
| ASV 72* Faecalimonas umbilicata | 0.03% | 0.01% | -3.4 | 3 / 12 | 1.7% | 0.7% | -2.3 | 4 / 9 |
| ASV 88 Akkermansia sp004167605 | 0.001% | 0.002% | 1.5 | 3 / 4 | 1.3% | 0.02% | -57.7 | 2 / 6 |
| ASV 29 Fusobacterium A | 0.000% | 0.002% | 4.9 | 3 / 3 | 2.0% | 3.0% | 1.5 | 4 / 7 |
| Note: ASVs included were those with a relative abundance over 1% in either group at T1 and T2. T1: Timepoint 1 (baseline); T2: Timepoint 2 (Post-intervention). The column titled "# of responders (% of total observed)": number of the responders to the change (positive or negative, indicated in "Fold Change"; The total observed indicates the total number of participants who were detected THE ASV. * ASV with * indicates the species was identified using the Basic Local Alignment Search Tool (BLAST) the online sequence database of National Centre for Biotechnology Information (NCBI). ¹ In ASV 11, 37: T1 between-group difference was significant, P < 0.01.  ² In ASV 18, 54: T1 between-group difference was significant, P < 0.05.  ³ In ASV 14, 37: T2 between- group difference was significant, P < 0.01.  Cell in bold indicates difference within ur gut® group was significant, P = 0.010.  All (False Discovery Rate) FDR.P > 0.05 | | | | | | | | |

Supplementary Table 15. Gut alpha diversity indices by group and timepoint

| Diversity indices | Group × Time Interaction P | ur gut® Group, n=13  Mean (SE) | | | Placebo Group, n=13  Mean (SE) | | | Between -group P |
| --- | --- | --- | --- | --- | --- | --- | --- | --- |
|  |  | T1 | T2 | T2-T1 | T1 | T2 | T2-T1 |  |
| Chao1 index¹ | 0.086 | 352 (18) | 340 (18) | -12 (20) | 274 (18) | 312 (18) | 38 (20) | 0.026* |
| Shannon index² | 0.207 | 3.9 (0.1) | 3.6 (0.1) | -0.3 (0.0) | 3.5 (0.1) | 3.4 (0.1) | 0.0 (0.1) | 0.122 |
| Fisher index³ | 0.068 | 48 (3) | 45 (3) | -2 (2) | 38 (3) | 41 (3) | 3 (2) | 0.047 |
| # of observed species⁴ | 0.075 | 343 (18) | 332 (18) | -11 (19) | 266 (18) | 304 (18) | 38 (19) | 0.026* |
| Note: the rarefied data was used throughout the analysis. T: timepoint. SE: Standard Error. Linear Mixed Modelling was used to assess differences between- and within-groups with BMI = 22.9625, and age = 37.15 included as covariates in the model. *FDR-adjusted P =0.053.  Between-group difference at T1 (Placebo group ­ ur gut® group): ¹ -77 (26), P = 0.006; ² -0.4 (0.2), P = 0.052; ³-10 (4), P = 0.011; ⁴ -78 (26), P = 0.005 | | | | | | | | |

**References**

1.	Ohayon M, Wickwire EM, Hirshkowitz M, et al. National Sleep Foundation's sleep quality recommendations: first report. Sleep Health 2017;3:6-19.

2.	Drossman DA, Hasler WL. Rome IV-Functional GI Disorders: Disorders of Gut-Brain Interaction. Gastroenterology 2016;150:1257-61.

3.	McRorie JW, Gibb RD, Sloan KJ, McKeown NM. Psyllium. Nutrition Today 2021;56:169-182.

4.	Zarrin R, Ibiebele TI, Marks GC. Development and validity assessment of a diet quality index for Australians. Asia Pac J Clin Nutr 2013;22:177-87.

5.	Hlaing-Hlaing H, Pezdirc K, Tavener M, et al. Diet quality indices used in Australian and New Zealand adults: A systematic review and critical appraisal. Nutrients 2020;12.

6.	National Health and Medical Research Council. Eat for Health: Australian Dietary Guidelines. In: Council NHaMR, ed. Canberra, Australia, 2013.

7.	Australian National Health and Medical Research Council (NHMRC). Nutrient Reference Values for Australia and New Zealand (nrv.gov.au). 22/09/2017 ed, 2017.

8.	Stinson LF, Boyce MC, Payne MS, Keelan JA. The not-so-sterile womb: Evidence that the human fetus is exposed to bacteria prior to birth. Front Microbiol 2019;10:1124.

9.	Jones J, Reinke SN, Ali A, et al. Fecal sample collection methods and time of day impact microbiome composition and short chain fatty acid concentrations. Sci Rep 2021;11:13964.

10.	Martin M. Cutadapt removes adapter sequences from high-throughput sequencing reads. EMBnet.journal 2011;17:10-12.

11.	Callahan BJ, McMurdie PJ, Rosen MJ, et al. DADA2: High-resolution sample inference from Illumina amplicon data. Nature methods 2016;13:581-3.

12.	Parks DH, Chuvochina M, Waite DW, et al. A standardized bacterial taxonomy based on genome phylogeny substantially revises the tree of life. Nature biotechnology 2018;36:996-1004.

13.	Birkett AM, Jones GP, deSilva AM, et al. Dietary intake and faecal excretion of carbohydrate by Australians: importance of achieving stool weights greater than 150 g to improve faecal markers relevant to colon cancer risk. European Journal of Clinical Nutrition 1997;51:625-632.

14.	Microsoft Corporation. Microsoft Excel. 2102 ed. Redmond, WA, 2018.

15.	IBM. IBM SPSS Statistics for Windows. 25.0 ed. Armonk, NY: IBM Corp., 2017.
